# A parametric similarity measure for neutrosophic set and its applications in energy production

**DOI:** 10.1016/j.heliyon.2024.e38272

**Published:** 2024-09-30

**Authors:** Peide Liu, Muhammad Azeem, Mehwish Sarfraz, Senesie Swaray, Bandar Almohsen

**Affiliations:** a School of Business Administration, Shandong Women's University, Shandong, Jinan, 250300, China; b School of Management Science and Engineering, Shandong University of Finance and Economics, Jinan, Shandong, 250014, China; c Department of Mathematics, Riphah International University, Lahore, 54000, Pakistan; d Independent Researcher, Lahore, Pakistan; e Tree Crops Unit, Sierra Leone Agricultural Research Institute, Freetown, Sierra Leone; f Department of Mathematics, College of Science, King Saud University, P.O. Box 2455, Riyadh, 11451, Saudi Arabia

**Keywords:** Single value neutrosophic set, Similarity measure, Multi-attribute decision-making, Energy production

## Abstract

As a useful tool for managing ambiguous and inconsistent data, the Single Value Neutrosophic Set (SVNSs) is an extension of both Fuzzy Sets (FSs) and Intuitionistic Fuzzy Sets (IFSs). In the field of information theory, metrics like similarity, entropy, and distance are important. Although a number of entropy measures for SVNSs have been put forth and used in real-world situations, both academic research and real-world applications have pointed out certain drawbacks. Additionally, the Similarity Measures (SMs) is a useful instrument for determining how similar any two fuzzy values are to one another. The distance between the values allows the current SMs to evaluate the similarity. However, due to a few characteristics and intricate value operations, there are irrational and nonsensical cases. To deal with these preposterous cases, this paper proposed a parametric similarity measure in view of three parameters $m_{1},m_{2},m_{3}$ in which decision makers can obtain the appropriate SMs by changing parameters with different decision styles. Furthermore, we analyze some existing SMs from a mathematical perspective and demonstrate the success of the proposed SMs using mathematical models. Ultimately, we apply the suggested SMs to resolve the Multi-Attribute Decision-Making (MADM) problems. We learn from the correlation and analysis that the suggested SM outperforms certain other SMs that are based on the SVNSs.

## 1 Introduction

Single value Neutrosophic sets [1] (SVNSs) offer a structure for handling ambiguity, imprecision, and insufficient data. It is common for information to be imprecise in the real world, and conventional sets might not be able to fully represent this uncertainty. Sometimes there may be gaps in the knowledge about a set's elements, and SVNSs allows for the representation of unknown values or partial knowledge. This is especially important when making decisions because it's common to not have all the information needed. When there is ambiguity or imprecision in defining an element's membership in a set, SVNSs can be used to symbolize that ambiguity. This is especially important when there are ambiguous boundaries between categories.

SVNSs are an extension of traditional Fuzzy Sets (FSs) [2], Intuitionistic Fuzzy Sets [3] (IFSs). IFSs take into account indeterminacy and add the non-membership degree, in contrast to FSs, which only have a membership degree that expresses determinacy. The membership degree and non-membership degree were extended to interval numbers by Atanassov [4], who also proposed the interval-valued IFS (IVIFS). Nonetheless, there are situations in which the membership degree and non-membership degree are unable to clearly convey hazy information. Therefore, Neutrosophic sets (NSs) were introduced by Smarandache in 1998 [5]. Three membership functions a membership degree (MD), a non-membership degree (NMD), and an abstinence degree (AD) define NS within a universal set X. The neutrosophic component degree MD(μ),AD(μ), and ND(μ) for every element μ in X are real standard or nonstandard subsets of the interval ]−0,1+[. On the other hand, these elements present difficulties when used to solve practical issues. Moreover, a great deal of theories concerning neutrosophic sets are progressively proposed. Ye [6], for instance, proposed a subset of NS called the simple neutrosophic set (SNS). In response to this difficulty, Wang et al. [1] introduced the SVNSs, which offer a more useful and controllable representation for solving problems in the real world. Regarding SVNSs, the three memberships, MD(μ),AD(μ), and ND(μ) are real standard subsets of the interval [0,3] for each element μ in the universal set X. SVNSs [7] generalize IFSs, inconsistent IFSs (Picture Fuzzy Sets, Ternary Fuzzy Sets), Pythagorean Fuzzy Sets (PyFs), q-Rung Orthopair Fuzzy Sets (q-ROFs), Spherical Fuzzy Sets (SFs), and n-Hyper Spherical Fuzzy Sets (n-HSFs). SVNSs, which have been applied to various real-world problems and studied theoretically, are designed to handle incomplete, inconsistent, and uncertain information [[8], [9], [10]].

### 1.1 Literature review

In order to extend the aggregation operators to SVNSs and combine Hamacher operations, Ye looked into different ways to measure the similarity of SVNSs and used them in the fields of engineering, cluster analysis, and MADM [11]. This is in contrast to many of the similarity measures mentioned above, which are either generated from distance measures or directly determined by explicit formulas. Ye, J [12]. introduced the concept of similarity measures on SVNSs and applied it to MADM [13]. Given the concept of similarity measures on SVNSs using the application of MADM. Ye, J [14]. expend the theory of similarity measures on SVNSs use the applications on medical diagnoses. Ali et al. [15] given the concept of complex neutrosophic on Dic similarity measures and use the application of MADM. Liu et al. [16] proposed several new aggregation operators that could be used in MADM. Saqlain et al. [17] develop the concept of similarity measures for SVNSs with TOPSIS method and use the application of MADM. Ye et al. [18] given the concept of interval value Similarity measure on SVNSs [19]. Given the concept of distance N Z-number sets and use the application of MADM. Chai et al. [20] expanded the concepts of NS using the similarity measure on MADM with medical diagnosis. Peng et al. [21] developed the theory of similarity measure on SVNSs with industry evaluation. Sahin et al. [22] given the concept of single value SVNSs use the application of medical with similarity measure. Sarfraz et al. [23] introduced the SMs on spherical fuzzy. Bui et al. [24] expanded the theory on similarity measure with NS. The theory on SVNSs was developed by Thao et al. [25] using similarity measures with MADM. By utilizing MADM, Ali et al. [26] presented the idea of similarity measures on SVNSs. Ozlu et al. [27] developed the concept of SVNSs type-2 fuzzy set and use the application of TOPSIS. A novel idea of similarity measures within the complex SVNSs environment was presented by Mondal et al. [28].

Borah et al. [29] developed the theory of MADM with convex combination weighted vector on similarity measure on NS. The theory of SVNSs was introduced by Mandour et al. [30] to address image processing problems. Chaira et al. [31] expand the theory of SVNSs on segmenting abnormal regions in mammogram. Bakro et al. [32] given the concept of SVNSs with use the application of digital images. Kaur et al. [33] given the concept of SVNSs generalized linguistic cubic aggregation operator. Poonia et al. [34] given the concept of neutrosophic hesitant on PFS with MADM. The idea of an intelligent detection technique for forging defect detection was first presented by Tang Yu et al. [35,36]. A novel idea for NS tangent measures in MADM was created by K Mondal et al. [37]. El-Shorbagy [38] expand the theory of SVNSs with image enhancement. Song et al. [39] develop the theory of SVNSs with segmentation algorithm on salience map. Thong et al. [40] expand the theory of PN semi-supervised fuzzy clustering for noisy data. Mandour et al. [41] Dey et al. [42] expand the theory of SVNSs with different types of graphs operations. Using a bio-inspired algorithm, Yan et al. [[43], [44], [45]] developed the theory of a fuel cell hybrid microgrid system. Jafar et al. [46] given the new concepys o Prioritized Aggregation Operators. SVNSs on trigonometric with similarity measures. Sarfraz [[47], [48], [49], [50], [51]] introduced the theory of Schweizer-Sklar Chou et al. [52] given the concept of interval value SVNSs use the application of energy selection with dis similarity measure. Froglu et al. [53] develop the theory of SVNSs on MADM with distance measure. Sarfraz et al. introduced the theory of [[54], [55], [56], [57]] Aczel-Alsina aggregation operator. In MADM in NSs, Pramanik et al. [58] developed the theory of similarity measures.

[59,60] introduce the SMs for the framework of IFSs and discussed the application of the developed SMs to the pattern recognition [61]. introduced the SMs for qROFS, and [62] introduced the SMs for PyFS and discussed the interesting applications of that SMs. Wei [63] introduced the cosine SMs (CSMs) and contingent similarity measures (CSMs) base of the cosine function and contingent function respectively for the PFS and applied them to the MADM. Wei and Geo [64] developed Dice SM for the PFS. Dinh and Thao [65] introduced the some SMs for PFS and applied to the problem of MADM. Singh and Mishre [66] extended the SMs by considering the refusal degree of the PFS and applied the SMs to the problem of the clustering. Luo and Zhang [67] introduce the SMs based on some basic operations for the PFS.

### 1.2 Motivation of the research

From the overview above, we can see that different SMs can be applied to real-world MADM problems. The evaluation data should be handled more quickly and readily in order to use the MADM method to find the best option. One of the most efficient methods for performing an aggregation function is the SVNSs, which is a generalized form of IFs, PYFs, and q-ROFS. As with MSM and MM, HM can show how different attributes are related to one another, but its computation is not as involved. In the SVNSs context, the SMs operator hasn't been expanded, though. Furthermore, SMs is more adaptable and generalized than other operations, like Frank, Einstein, and Hamcher. Although it can currently be combined with different aggregation functions, research on the new SMs which is based on a SVNSs and has the ability to capture the interrelationships among multiple variables has sadly not yet surfaced. The development of a number of SVNSs based on the benefits of SMs and the creation of some aggregation function-based MADM methods in the SVNSs environment are required to close the aforementioned two research gaps.

### 1.3 Aim of the study

Developing and studying a parametric similarity measure for SVNSs is the goal of this research, with a focus on its potential applications in the energy production industry. This work aims to further SVNSs theory by presenting a new similarity metric and investigating its applicability in solving problems in the field of energy production. Create and define a parametric similarity measure specifically for SVNSs, taking into account important variables that affect the measure's effectiveness. Provide a theoretical framework, examine the mathematical characteristics of the suggested parametric similarity measure, and show how well it captures the relationships between SVNSs. Incorporate the created similarity measure into models of energy production and evaluate its effects on decision-making, system optimization, and overall performance relative to other similarity measures already in use.

### 1.4 Contribution

Some conclusions from the SMs discussed above in the form of the key points are given below.

• All the SMs for the IFSs, PYFS, qROFS, and PFS are outdate because these frameworks can extract very limited information from the real-life scenarios. Hence, the decisions makers cannot find the best results due to the involvement of the uncertainty and the information loss. Hence, the advanced SMs should be defined for the SVNSs that can assess the similarity between pictures with less uncertainty.
• Some of the SMs discussed above are failed to compute at some special scenarios. For example, some of the SMs do not provide the decision results due to the division by zero problems. Therefore, to improve the identification ability of the SMs and overcome the defects of current SMs, it is very necessary to propose a new SMs.

### 1.5 Organization of the study

In this paper, first some basic concepts are discussed in section 2. In section 3, used the parameter are introduced to solve the rejected information then the new SVNSs can be generated. Decision makers can be used of different decision styles can find the appropriate similarity measure by selection the parameters $m_{1},m_{2},m_{3}$. But the similarity measure effectiveness illustrated by multi attribute decision making and numerical examples.

## 2 Preliminaries

This section explains a few fundamental SVNSs concepts. We also restate the concept of the entropy and similarity measure of the SVNSs in the different settings.

Definition 1 [7]: On a set X a SVNSs is of the shape $=\left\{\left(\mu ,\left(\lambda \;\left(\mu \right),{\vartheta \;\left(\mu \right),d}^{\prime }\;\left(\mu \right)\right)\right)|\;\mu \in X\right\}$ . λ(μ)∈[0,1] Is called membership degree of I, ϑ(μ)∈[0,1] is called abstinence degree of I and $d^{\prime }\in \left[0,1\right]$ is called non-membership degree of I and satisfy the following condition $0\leq Sum\left(\lambda \left(\mu \right),\vartheta \left(\mu \right){,d}^{\prime }\left(\mu \right)\right)\leq 3$. Further, $r\left(\mu \right)=1-sum\left(\lambda \left(\mu \right),\vartheta \left(\mu \right){,d}^{\prime }\left(\mu \right)\right)$ represents the refusal degree of μ∈X and the pair $\left(\lambda ,\vartheta ,d^{\prime }\right)$ is termed as a SVNSs.

Definition 2 [7]: Let $A=\left\{\left(\mu ,\lambda_{A}\left(\mu_{\tau }\right),\vartheta_{A}\left(\mu_{\tau }\right),d_{A}^{\prime }\left(\mu_{\tau }\right)\right)│\mu \in X\right\}$ and $B=\left\{\left(\mu ,\lambda_{B}\left(\mu_{\tau }\right),\vartheta_{B}\left(\mu_{\tau }\right),d_{B}^{\prime }\left(\mu_{\tau }\right)\right)│\mu \in X\right\}$ be any two be SVNSs on universe X, then

1. A⊆B If and only if $\lambda_{A}\left(\mu_{\tau }\right)\leq \lambda_{B}\left(\mu_{\tau }\right)$, $\vartheta_{A}\left(\mu_{\tau }\right)\geq \vartheta_{B}\left(\mu_{\tau }\right)$, $d_{A}^{\prime }\left(\mu_{\tau }\right)\geq d_{B}^{\prime }\left(\mu_{\tau }\right)$. For all μϵX.
2. A=B If and only if A⊆B and B⊆A i.e. $\lambda_{A}\left(\mu_{\tau }\right)=\lambda_{B}\left(\mu_{\tau }\right)$, $\vartheta_{A}\left(\mu_{\tau }\right)=\vartheta_{B}\left(\mu_{\tau }\right)$, $d_{A}^{\prime }\left(\mu_{\tau }\right)=d_{B}^{\prime }\left(\mu_{\tau }\right)$.
3. $A^{c}=\left\{\left(\mu ,{1-\lambda }_{A}\left(\mu_{\tau }\right),{1-\vartheta }_{A}\left(\mu_{\tau }\right),d_{A}^{\prime }\left(\mu_{\tau }\right)\right)|\mu \in X\right\}$.

Definition 3 [68]: Let $A=\left\{\left(\mu ,\lambda_{A}\left(\mu_{\tau }\right),\tau_{A}\left(\mu_{\tau }\right),d_{A}^{\prime }\left(\mu_{\tau }\right)\right)│\mu \in X\right\}$ and $B=\left\{\left(\mu ,\lambda_{B}\left(\mu_{\tau }\right),\tau_{B}\left(\mu_{\tau }\right),d_{B}^{\prime }\left(\mu_{\tau }\right)\right)│\mu \in X\right\}$ be any two be SVNSs on universe X, then the SM between A and B is defined as L(A,B), which satisfies the following axioms:

• $\left(L_{1}\right)\;0\leq L\left(A,B\right)\leq 1$**;**
• $\left(L_{2}\right)\;L\left(A,B\right)=1$ iff A=B**;**
• $\left(L_{3}\right)\;L\left(A,B\right)=L\left(B,A\right)$**;**
• $\left(L_{4}\right)$ Let C be any SVNSs such that A⊆B⊆C, then L(A,C)≤L(A,B) and L(A,C)≤L(B,C)**;**

Now, we review some existing similarity measures between SVNSs in the following.

Let $A=\left\{\left(\mu_{\tau },\lambda_{A}\left(\mu_{\tau }\right),\vartheta_{A}\left(\mu_{\tau }\right),d_{A}^{\prime }\left(\mu_{\tau }\right)\right)│\mu_{\tau }\in X\right\}$ and $B=\left\{\left(\mu_{\tau },\lambda_{B}\left(\mu_{\tau }\right),\vartheta_{B}\left(\mu_{\tau }\right),d_{B}^{\prime }\left(\mu_{\tau }\right)\right)│\mu_{\tau }\in X\right\}$ be any two be SVNSs on $X=\left\{\mu_{1},\mu_{2},\ldots ,\mu_{n}\right\}$ , $\rho_{A}\left(\mu_{\tau }\right)$ and $\rho_{B}\left(\mu_{\tau }\right)$ be the refusal degrees of element $\mu_{\tau }$ belonging to SVNSs A and B respectively, where $\rho_{A}\left(\mu_{\tau }\right)=1-\lambda_{A}^{n}\left(\mu_{\tau }\right),\vartheta_{A}^{n}\left(\mu_{\tau }\right),d_{A}^{\prime }\left(\mu_{\tau }\right)$ and $\rho_{B}\left(\mu_{\tau }\right)=1-\lambda_{B}\left(\mu_{\tau }\right),\vartheta_{B}^{n}\left(\mu_{\tau }\right),d_{B}^{\prime }\left(\mu_{\tau }\right)$ . The existing similarity degrees between SVNSs A and B are reviewed as follows:

Where τ=1,2,3…n.

The SMs defined by Ref. [69] for the based on the SVNSs is given as follows given in Equations (1), (2), (3).

(1) $$L_{1}\left(A,B\right)=1-\frac{1}{3n}\sum_{\tau =1}^{n}\left(│\lambda_{A}\left(\mu_{\tau }\right)-\lambda_{B}\left(\mu_{\tau }\right)│+│\vartheta_{A}\left(\mu_{\tau }\right)-\vartheta_{B}\left(\mu_{\tau }\right)│+│d_{A}^{\prime }\left(\mu_{\tau }\right)-d_{B}^{\prime }\left(\mu_{\tau }\right)│\right)$$

(2) $$L_{2}\left(A,B\right)=1-\frac{1}{3n}\sum_{\tau =1}^{n}\left(│\lambda_{A}\left(\mu_{\tau }\right)-\lambda_{B}\left(\mu_{\tau }\right){│}^{2}+│\vartheta_{A}\left(\mu_{\tau }\right)-\vartheta_{B}\left(\mu_{\tau }\right){│}^{2}+│d_{A}^{\prime }\left(\mu_{\tau }\right)-d_{B}^{\prime }\left(\mu_{\tau }\right){│}^{2}\right)$$

(3) $$L_{3}\left(A,B\right)=\frac{1-\frac{1}{3n}\sum_{\tau =1}^{n}\left(│\lambda_{A}\left(\mu_{\tau }\right)-\lambda_{B}\left(\mu_{\tau }\right)│+│\vartheta_{A}\left(\mu_{\tau }\right)-\vartheta_{B}\left(\mu_{\tau }\right)│+│d_{A}^{\prime }\left(\mu_{\tau }\right)-d_{B}^{\prime }\left(\mu_{\tau }\right)│\right)}{1+\frac{1}{3n}\sum_{\tau =1}^{n}\left(│\lambda_{A}\left(\mu_{\tau }\right)-\lambda_{B}\left(\mu_{\tau }\right)│+│\vartheta_{A}\left(\mu_{\tau }\right)-\vartheta_{B}\left(\mu_{\tau }\right)│+│d_{A}^{\prime }\left(\mu_{\tau }\right)-d_{B}^{\prime }\left(\mu_{\tau }\right)│\right)}$$

Cosine Function which are Equations (4), (5) [14].

(4) $$L_{4}\left(A,B\right)=\frac{1}{n}\sum_{\tau =1}^{n}co\lambda \;\left(\frac{\pi }{2}ma\mu \left(│\lambda_{A}\left(\mu_{\tau }\right)-\lambda_{B}\left(\mu_{\tau }\right)│,│\vartheta_{A}\left(\mu_{\tau }\right)-\vartheta_{B}\left(\mu_{\tau }\right)│,│d_{A}^{\prime }\left(\mu_{\tau }\right)-d_{B}^{\prime }\left(\mu_{\tau }\right)│\right)\right)$$

(5) $$L_{5}\left(A,B\right)=\frac{1}{n}\sum_{\tau =1}^{n}co\lambda \;\left(\frac{\pi }{6}Sum\left(│\lambda_{A}\left(\mu_{\tau }\right)-\lambda_{B}\left(\mu_{\tau }\right)│,│\vartheta_{A}\left(\mu_{\tau }\right)-\vartheta_{B}\left(\mu_{\tau }\right)│,│d_{A}^{\prime }\left(\mu_{\tau }\right)-d_{B}^{\prime }\left(\mu_{\tau }\right)│\right)\right)$$

Tangent Similarity Function which are Equations (6), (7) [70].

(6) $$L_{6}\left(A,B\right)=1-\frac{1}{n}\sum_{\tau =1}^{n}\;\tan\;\left(\frac{\pi }{4}ma\mu \left(│\lambda_{A}\left(\mu_{\tau }\right)-\lambda_{B}\left(\mu_{\tau }\right)│,│\vartheta_{A}\left(\mu_{\tau }\right)-\vartheta_{B}\left(\mu_{\tau }\right)│,│d_{A}^{\prime }\left(\mu_{\tau }\right)-d_{B}^{\prime }\left(\mu_{\tau }\right)│\right)\right)$$

(7) $$L_{7}\left(A,B\right)=1-\frac{1}{n}\sum_{\tau =1}^{n}\;\tan\;\left(\frac{\pi }{12}Sum\left(│\lambda_{A}\left(\mu_{\tau }\right)-\lambda_{B}\left(\mu_{\tau }\right)│,│\vartheta_{A}\left(\mu_{\tau }\right)-\vartheta_{B}\left(\mu_{\tau }\right)│,│d_{A}^{\prime }\left(\mu_{\tau }\right)-d_{B}^{\prime }\left(\mu_{\tau }\right)│\right)\right)$$

Cotangent Similarity Function which are Equations (8), (9) [71].

(8) $$L_{8}\left(A,B\right)=\frac{1}{n}\sum_{\tau =1}^{n}\;\cot\;\left(\frac{\pi }{4}+\frac{\pi }{4}ma\mu \left(│\lambda_{A}\left(\mu_{\tau }\right)-\lambda_{B}\left(\mu_{\tau }\right)│,│\vartheta_{A}\left(\mu_{\tau }\right)-\vartheta_{B}\left(\mu_{\tau }\right)│,│d_{A}^{\prime }\left(\mu_{\tau }\right)-d_{B}^{\prime }\left(\mu_{\tau }\right)│\right)\right)$$

(9) $$L_{9}\left(A,B\right)=\frac{1}{n}\sum_{\tau =1}^{n}\;\cot\;\left(\frac{\pi }{4}+\frac{\pi }{12}Sum\left(│\lambda_{A}\left(\mu_{\tau }\right)-\lambda_{B}\left(\mu_{\tau }\right)│,│\vartheta_{A}\left(\mu_{\tau }\right)-\vartheta_{B}\left(\mu_{\tau }\right)│,│d_{A}^{\prime }\left(\mu_{\tau }\right)-d_{B}^{\prime }\left(\mu_{\tau }\right)│\right)\right)$$

Similarity Function which is Equation (10) [18].

(10) $$L_{10}\left(A,B\right)=1-\frac{1}{n}\sum_{\tau =1}^{n}\;\left(ma\mu \left(│\lambda_{A}\left(\mu_{\tau }\right)-\lambda_{B}\left(\mu_{\tau }\right)│,│\vartheta_{A}\left(\mu_{\tau }\right)-\vartheta_{B}\left(\mu_{\tau }\right)│,│d_{A}^{\prime }\left(\mu_{\tau }\right)-d_{B}^{\prime }\left(\mu_{\tau }\right)│\right)\right)$$

Cosine Similarity Measure which is Equation (11) [11].

(11) $$L_{11}\left(A,B\right)=\frac{1}{n}\sum_{\tau =1}^{n}\frac{\left(\lambda_{A}\left(\mu_{\tau }\right).\lambda_{B}\left(\mu_{\tau }\right)\right)+\left(\vartheta_{A}\left(\mu_{\tau }\right).\vartheta_{B}\left(\mu_{\tau }\right)\right)+\left(d_{A}^{\prime }\left(\mu_{\tau }\right).d_{B}^{\prime }\left(\mu_{\tau }\right)\right)}{\sqrt{{\left(\lambda_{A}\left(\mu_{\tau }\right)\right)}^{2}+{\left(\vartheta_{A}\left(\mu_{\tau }\right)\right)}^{2}+{\left({d^{\prime }}_{A}\left(\mu_{\tau }\right)\right)}^{2}}\sqrt{{\left(\lambda_{B}\left(\mu_{\tau }\right)\right)}^{2}+{\left(\vartheta_{B}\left(\mu_{\tau }\right)\right)}^{2}+{\left({d^{\prime }}_{B}\left(\mu_{\tau }\right)\right)}^{2}}}$$

Dice Similarity Measure which are Equations (12), (13) [14].

(12) $$L_{12}\left(A,B\right)=\frac{1}{n}\sum_{\tau =1}^{n}\frac{2\left(\left(\lambda_{A}\left(\mu_{\tau }\right).\lambda_{B}\left(\mu_{\tau }\right)\right)+\left(\vartheta_{A}\left(\mu_{\tau }\right).\vartheta_{B}\left(\mu_{\tau }\right)\right)+\left(d_{A}^{\prime }\left(\mu_{\tau }\right).d_{B}^{\prime }\left(\mu_{\tau }\right)\right)\right)}{\left({\left(\lambda_{A}\left(\mu_{\tau }\right)\right)}^{2}+{\left(\tau_{A}\left(\mu_{\tau }\right)\right)}^{2}+{\left({d^{\prime }}_{A}\left(\mu_{\tau }\right)\right)}^{2}\right)+\left({\left(\lambda_{B}\left(\mu_{\tau }\right)\right)}^{2}+{\left(\tau_{B}\left(\mu_{\tau }\right)\right)}^{2}+{\left({d^{\prime }}_{B}\left(\mu_{\tau }\right)\right)}^{2}\right)}$$

(13) $$L_{13}\left(A,B\right)=\frac{1}{n}\sum_{\tau =1}^{n}\frac{\left(\left(\lambda_{A}\left(\mu_{\tau }\right).\lambda_{B}\left(\mu_{\tau }\right)\right)+\left(\vartheta_{A}\left(\mu_{\tau }\right).\vartheta_{B}\left(\mu_{\tau }\right)\right)+\left(d_{A}^{\prime }\left(\mu_{\tau }\right).d_{B}^{\prime }\left(\mu_{\tau }\right)\right)\right)}{\left({\left(\lambda_{A}\left(\mu_{\tau }\right)\right)}^{2}+{\left(\vartheta_{A}\left(\mu_{\tau }\right)\right)}^{2}+{\left({d^{\prime }}_{A}\left(\mu_{\tau }\right)\right)}^{2}+{\left(\lambda_{B}\left(\mu_{\tau }\right)\right)}^{2}+{\left(\vartheta_{B}\left(\mu_{\tau }\right)\right)}^{2}+{\left({d^{\prime }}_{B}\left(\mu_{\tau }\right)\right)}^{2}\right)-\left(\left(\lambda_{A}\left(\mu_{\tau }\right).\lambda_{B}\left(\mu_{\tau }\right)\right)+\left(\vartheta_{A}\left(\mu_{\tau }\right).\vartheta_{B}\left(\mu_{\tau }\right)\right)+\left(d_{A}^{\prime }\left(\mu_{\tau }\right).d_{B}^{\prime }\left(\mu_{\tau }\right)\right)\right)}$$

## 3 An analysis of some existing single value neutrosophic similarity measures

Using the SMs as a numerical tool to determine the degree of similarity between objects, decision-making, clinical determination, and example acknowledgment problems have all been resolved. Despite the fact that many SMs between SVNSs have been proposed, their practical application can lead to irrational and counterintuitive outcomes that pose significant challenges for functional clients. In the following, we present a thorough analysis of a few of the SMs that are currently in use from an arithmetic perspective, as shown in Table 1.

**Table 1:** A comprehensive analysis of some existing Similarity Measures for SVNSs.

L	**Does not meet the axiom**L2	**The division by zero problem**	**Serious information loss**
$L_{1}$	NO	NO	NO
$L_{2}$	NO	NO	NO
$L_{3}$	NO	NO	yes
$L_{4}$	NO	NO	NO
$L_{5}$	NO	NO	NO
$L_{6}$	NO	NO	NO
$L_{7}$	NO	NO	NO
$L_{8}$	Yes	NO	Yes
$L_{9}$	Yes	NO	Yes
$L_{10}$	NO	NO	NO
$L_{11}$	NO	NO	NO
$L_{12}$	NO	NO	NO
$L_{13}$	NO	NO	NO
$L_{14}$	NO	NO	NO
$L_{m}$	Yes	NO	NO

The axiom $L_{2}$ is one of the most basic axioms of SVNSs SMs. By analyzing Table 1, we can easily find that the similarity measures $L_{7}$ and $L_{8}$ do not satisfy this axiom. The detailed discussion is as follows:

(1). Let $A=\left\{\left(\mu_{\tau },\lambda_{A}\left(\mu_{\tau }\right),\vartheta_{A}\left(\mu_{\tau }\right),d_{A}^{\prime }\left(\mu_{\tau }\right)\right)│\mu_{\tau }\in X\right\}$ and $B=\left\{\left(\mu_{\tau },\lambda_{B}\left(\mu_{\tau }\right),\vartheta_{B}\left(\mu_{\tau }\right),d_{B}^{\prime }\left(\mu_{\tau }\right)\right)│\mu_{\tau }\in X\right\}$ be any two be SVNSs on $X=\left\{\mu_{1},\mu_{2},\ldots ,\mu_{n}\right\}$. For the similarity measure $L_{8}$ and $L_{9}$, there are two cases in which does not satisfy the axiom $\left(L_{2}\right)$ L(A,B) = 1 implies A=B as shown below:
(2). The similar SMs $L_{3}$, $L_{8}\text{,}$ and $L_{9}$ do not satisfy the axiom $\left(L_{1}\right)\;0\leq L\left(A,B\right)\leq 1$**;** and these SMs provide a counter-intuitive result for practical users in this case.

## 4 An assessment of the parametric similarity between single value neutrosophic sets

In order to get around the limitations of the current SMs, we expand a parametric SMs between SVNSs in this section, taking into consideration the explanations for the absurd results of the analysis presented in Table 1. In this section, we develop a paired capability to provide a parametric SVNS of SMs. According to the analysis in Table 1 the SMs $L_{3}$, $L_{8}\text{,}$ and $L_{9}$ have the drawbacks. Hence, the parametric SMs are introduced in following in Definition 8. We only take into account the outcomes produced by the suggested and current similarity measures $L_{1}$, $L_{2}\text{,}$ $L_{4}$, $L_{5},L_{6}$, $L_{7},L_{10}$, ${\;L}_{11},L_{12}$ and $L_{13}$.

Definition 4 Let $A=\left\{\left(\mu_{\tau },\lambda_{A}\left(\mu_{\tau }\right),\tau_{A}\left(\mu_{\tau }\right),d_{A}^{\prime }\left(\mu_{\tau }\right)\right)│\mu_{\tau }\in X\right\}$ and $B=\left\{\left(\left(\mu_{\tau },\lambda_{B}\left(\mu_{\tau }\right),\tau_{B}\left(\mu_{\tau }\right),d_{B}^{\prime }\left(\mu_{\tau }\right)\right)\right)│\mu_{\tau }\in X\right\}$ be any two be SVNSs on $X=\left\{\mu_{1},\mu_{2},\ldots ,\mu_{n}\right\}$ then the function $L_{m}:SVNSs\left(\mu \right)\times SVNSs\left(\mu \right)\to \left[0,3\right]$ defined by Equation (14)

(14) $$L_{m}\left(A,B\right)=1-{\left[\frac{1}{3n}\sum_{\tau =1}^{n}\Delta_{1\;AB}^{p}\left(\mu_{\tau }\right)+\Delta_{2\;AB}^{p}\left(\mu_{\tau }\right)+\Delta_{3\;AB}^{p}\left(\mu_{\tau }\right)\right]}^{\frac{1}{p}}$$

$L_{m}\left(A,B\right)$ Is a similarity measure between A and B, where,

$$\Delta_{1\;AB}\left(\mu_{\tau }\right)=\frac{1}{m_{1}+1}│m_{1}\left(\lambda_{A}\left(\mu_{\tau }\right)-\lambda_{B}\left(\mu_{\tau }\right)\right)-\left(\vartheta_{A}\left(\mu_{\tau }\right)-\vartheta_{B}\left(\mu_{\tau }\right)\right)-\left(d_{A}^{\prime }\left(\mu_{\tau }\right)-d_{B}^{\prime }\left(\mu_{\tau }\right)\right)│m_{1}\in [0,+\infty )\text{,}$$

$$\Delta_{2\;AB}\left(\mu_{\tau }\right)=\frac{1}{2m_{2}+1}│m_{2}\left(\vartheta_{A}\left(\mu_{\tau }\right)-\vartheta_{B}\left(\mu_{\tau }\right)\right)-\left(\lambda_{A}\left(\mu_{\tau }\right)-\lambda_{B}\left(\mu_{\tau }\right)\right)-\left(d_{A}^{\prime }\left(\mu_{\tau }\right)-d_{B}^{\prime }\left(\mu_{\tau }\right)\right)│m_{2}\in [0,+\infty )\text{,}$$

$$\Delta_{3\;AB}\left(\mu_{\tau }\right)=\frac{1}{2m_{3}+1}│m_{3}\left(d_{A}^{\prime }\left(\mu_{\tau }\right)-d_{B}^{\prime }\left(\mu_{\tau }\right)\right)-\left(\lambda_{A}\left(\mu_{\tau }\right)-\lambda_{B}\left(\mu_{\tau }\right)\right)-\left(\vartheta_{A}\left(\mu_{\tau }\right)-\vartheta_{B}\left(\mu_{\tau }\right)\right)│m_{3}\in [0,+\infty )\text{,}$$

$\frac{1}{m_{1}+1}+\frac{1}{{2m}_{2}+1}+\frac{1}{{2m}_{3}+1}\in \left[0,3\right]$, and p is any positive integer.

Theorem 1 *Let*$A=\left\{\left(\mu ,\lambda_{A}\left(\mu_{\tau }\right),\vartheta_{A}\left(\mu_{\tau }\right),d_{A}^{\prime }\left(\mu_{\tau }\right)\right)│\mu \in X\right\}$*and*$B=\left\{\left(\mu ,\lambda_{B}\left(\mu_{\tau }\right),\vartheta_{B}\left(\mu_{\tau }\right),d_{B}^{\prime }\left(\mu_{\tau }\right)\right)│\mu \in X\right\}$*be any two be SVNSs on universe*X, *then the SMs between*A*and*B*is defined as*$L_{m}\left(A,B\right)$, *which satisfies the following axioms*:

• $\left(L_{1}\right)\;0\leq L_{m}\left(A,B\right)\leq 3$.
• $\left(L_{2}\right)\;L_{m}\left(A,B\right)=1$*iff*A=B.
• $\left(L_{3}\right)\;L_{m}\left(A,B\right)=L_{m}\left(B,A\right)$.
• $\left(L_{4}\right)$*Let*C*be any SVNSs such that*A⊆B⊆C, *then*$L_{m}\left(A,C\right)\leq L_{m}\left(A,B\right)$*and*$L_{m}\left(A,C\right)\leq L_{m}\left(B,C\right)$.

***Proof*:***In order to prove that* Eq. (16) *is a SMs*, *we only need to prove* Eq. (14) *satisfies axioms* $\left(L_{1}\right)-\left(L_{4}\right)$ $A=\left\{\left(\mu_{\tau },\lambda_{A}\left(\mu_{\tau }\right),\vartheta_{A}\left(\mu_{\tau }\right),d_{A}^{\prime }\left(\mu_{\tau }\right)\right)│\mu_{\tau }\in X\right\}$ *and* $B=\left\{\left(\left(\mu_{\tau },\lambda_{B}\left(\mu_{\tau }\right),\vartheta_{B}\left(\mu_{\tau }\right),d_{B}^{\prime }\left(\mu_{\tau }\right)\right)\right)│\mu_{\tau }\in X\right\}$ *and* $C=\left\{\left(\left(\mu_{\tau },\lambda_{C}\left(\mu_{\tau }\right),\vartheta_{C}\left(\mu_{\tau }\right),d_{C}^{\prime }\left(\mu_{\tau }\right)\right)\right)│\mu_{\tau }\in X\right\}$ *be any three SVNSs on* $X=\left\{\mu_{1},\mu_{2},\ldots ,\mu_{n}\right\}$.

$\left(L_{1}\right)$*We can write the following equations*:

$$\Delta_{1\;AB}\left(\mu_{\tau }\right)=\frac{1}{m_{1}+1}│m_{1}\left(\lambda_{A}\left(\mu_{\tau }\right)-\lambda_{B}\left(\mu_{\tau }\right)\right)-\left(\vartheta_{A}\left(\mu_{\tau }\right)-\vartheta_{B}\left(\mu_{\tau }\right)\right)-\left(d_{A}^{\prime }\left(\mu_{\tau }\right)-d_{B}^{\prime }\left(\mu_{\tau }\right)\right)│m_{1}\in [0,+\infty )=\frac{1}{m_{1}+1}│\left(m_{1}\lambda_{A}\left(\mu_{\tau }\right)-\vartheta_{A}\left(\mu_{\tau }\right)-d_{A}^{\prime }\left(\mu_{\tau }\right)\right)-\left(m_{1}\lambda_{B}\left(\mu_{\tau }\right)-\vartheta_{B}\left(\mu_{\tau }\right)-d_{B}^{\prime }\left(\mu_{\tau }\right)\right)│$$

$$\Delta_{2\;AB}\left(\mu_{\tau }\right)=\frac{1}{2m_{2}+1}│m_{2}\left(\vartheta_{A}\left(\mu_{\tau }\right)-\vartheta_{B}\left(\mu_{\tau }\right)\right)-\left(\lambda_{A}\left(\mu_{\tau }\right)-\lambda_{B}\left(\mu_{\tau }\right)\right)+\left(d_{A}^{\prime }\left(\mu_{\tau }\right)-d_{B}^{\prime }\left(\mu_{\tau }\right)\right)│m_{2}\in [0,+\infty )=\frac{1}{2m_{2}+1}│\left(m_{2}\vartheta_{A}\left(\mu_{\tau }\right)-\lambda_{A}\left(\mu_{\tau }\right)-d_{A}^{\prime }\left(\mu_{\tau }\right)\right)-\left(m_{2}\vartheta_{B}\left(\mu_{\tau }\right)-\lambda_{B}\left(\mu_{\tau }\right)-d_{B}^{\prime }\left(\mu_{\tau }\right)\right)│$$

$$\Delta_{3\;AB}\left(\mu_{\tau }\right)=\frac{1}{2m_{3}+1}│m_{3}\left(d_{A}^{\prime }\left(\mu_{\tau }\right)-d_{B}^{\prime }\left(\mu_{\tau }\right)\right)-\left(\lambda_{A}^{n}\left(\mu_{\tau }\right)-\lambda_{B}\left(\mu_{\tau }\right)\right)+\left(\vartheta_{A}\left(\mu_{\tau }\right)-\vartheta_{B}\left(\mu_{\tau }\right)\right)│m_{3}\in [0,+\infty )=\frac{1}{2m_{3}+1}│\left({{m_{3}d}^{\prime }}_{A}\left(\mu_{\tau }\right)-\lambda_{A}\left(\mu_{\tau }\right)-\vartheta_{A}\left(\mu_{\tau }\right)\right)-\left(m_{3}d_{B}^{\prime }\left(\mu_{\tau }\right)-\lambda_{B}\left(\mu_{\tau }\right)-\vartheta_{B}\left(\mu_{\tau }\right)\right)│$$

*By*$\lambda_{A}\left(\mu_{\tau }\right),\vartheta_{A}\left(\mu_{\tau }\right),d_{A}^{\prime }\left(\mu_{\tau }\right),\lambda_{B}\left(\mu_{\tau }\right),\vartheta_{B}\left(\mu_{\tau }\right),d_{B}^{\prime }\left(\mu_{\tau }\right)\in \left[0,1\right]$*and*$\lambda_{A}\left(\mu_{\tau }\right)+\vartheta_{A}\left(\mu_{\tau }\right)+d_{A}^{\prime }\left(\mu_{\tau }\right)\leq 1\;\lambda_{B}\left(\mu_{\tau }\right)+\vartheta_{B}\left(\mu_{\tau }\right)+d_{B}^{\prime }\left(\mu_{\tau }\right)\leq 3$.

*We have*

$$-1\leq m_{1}\lambda_{A}\left(\mu_{\tau }\right)-\vartheta_{A}\left(\mu_{\tau }\right)-d_{A}^{\prime }\left(\mu_{\tau }\right)\leq m_{1}-m_{1}\leq -\left({m_{1}\lambda }_{B}\left(\mu_{\tau }\right)-\vartheta_{B}\left(\mu_{\tau }\right)-d_{B}^{\prime }\left(\mu_{\tau }\right)\right)\leq 1$$

$$0\leq │\left(m_{1}\lambda_{A}\left(\mu_{\tau }\right)-\vartheta_{A}\left(\mu_{\tau }\right)-d_{A}^{\prime }\left(\mu_{\tau }\right)\right)-\left(m_{1}\lambda_{B}\left(\mu_{\tau }\right)-\vartheta_{B}\left(\mu_{\tau }\right)-d_{B}^{\prime }\left(\mu_{\tau }\right)\right)│\leq m_{1}+1\;i.e,{0\leq \Delta }_{1\;AB}\left(\mu_{\tau }\right)\leq 1$$

*Then*

$$-1\leq m_{2}\vartheta_{A}\left(\mu_{\tau }\right)-\lambda_{A}\left(\mu_{\tau }\right)+d_{A}^{\prime }\left(\mu_{\tau }\right)\leq 1⋁m_{2}-\left(1⋁m_{2}\right)\leq -\left({m_{2}\vartheta }_{B}\left(\mu_{\tau }\right)-\lambda_{B}\left(\mu_{\tau }\right)+d_{B}^{\prime }\left(\mu_{\tau }\right)\right)\leq 1$$

*Similarly*, *we get the following inequalities*:

$$-1\leq m_{3}d_{A}^{\prime }\left(\mu_{\tau }\right)-\lambda_{A}\left(\mu_{\tau }\right)+\vartheta_{A}\left(\mu_{\tau }\right)\leq 1⋁m_{3}$$

$$-\left(1⋁m_{3}\right)\leq -\left(m_{3}d_{B}^{\prime }\left(\mu_{\tau }\right)-\lambda_{B}\left(\mu_{\tau }\right)+\vartheta_{B}\left(\mu_{\tau }\right)\right)\leq 1$$

*Then we obtain*:

$$0\leq │\left(m_{2}\vartheta_{A}\left(\mu_{\tau }\right)-\lambda_{A}\left(\mu_{\tau }\right)+d_{A}^{\prime }\left(\mu_{\tau }\right)\right)-\left({m_{2}\vartheta }_{B}\left(\mu_{\tau }\right)-\lambda_{B}\left(\mu_{\tau }\right)+d_{B}^{\prime }\left(\mu_{\tau }\right)\right)│\leq 2⋁m_{2}$$

$$0\leq │\left(m_{3}d_{A}^{\prime }\left(\mu_{\tau }\right)-\lambda_{A}\left(\mu_{\tau }\right)+\vartheta_{A}\left(\mu_{\tau }\right)\right)-\left(m_{3}d_{B}^{\prime }\left(\mu_{\tau }\right)-\lambda_{B}\left(\mu_{\tau }\right)+\vartheta_{B}\left(\mu_{\tau }\right)\right)│\leq 2⋁m_{3}$$

*It means that*:

$$0\leq \Delta_{2\;AB}\left(\mu_{\tau }\right)=\frac{1}{2m_{2}+1}│\left(m_{2}\vartheta_{A}\left(\mu_{\tau }\right)-\lambda_{A}\left(\mu_{\tau }\right)+d_{A}^{\prime }\left(\mu_{\tau }\right)\right)-\left({m_{2}\vartheta }_{B}\left(\mu_{\tau }\right)-\lambda_{B}\left(\mu_{\tau }\right)+d_{B}^{\prime }\left(\mu_{\tau }\right)\right)│\leq \frac{1}{m_{2}+1}⋁\frac{1}{2}\leq 1$$

$$0\leq \Delta_{3\;AB}\left(\mu_{\tau }\right)=\frac{1}{2m_{3}+1}│\left(m_{3}d_{A}^{\prime }\left(\mu_{\tau }\right)-\lambda_{A}\left(\mu_{\tau }\right)+\vartheta_{A}\left(\mu_{\tau }\right)\right)-\left(m_{3}d_{B}^{\prime }\left(\mu_{\tau }\right)-\lambda_{B}\left(\mu_{\tau }\right)+\vartheta_{B}\left(\mu_{\tau }\right)\right)│\leq \frac{1}{m_{3}+1}⋁\frac{1}{2}\leq 1$$

*Finally*, *we have*:

$$0\leq 1-{\left[\frac{1}{3n}\sum_{\tau =1}^{n}\Delta_{1\;AB}^{p}\left(\mu_{\tau }\right)+\Delta_{2\;AB}^{p}\left(\mu_{\tau }\right)+\Delta_{3\;AB}^{p}\left(\mu_{\tau }\right)\right]}^{\frac{1}{p}}\leq 1$$

*Therefore*,

$$\left(L_{1}\right)\;0\leq L_{m}\left(A,B\right)\leq 1$$

$\left(L_{2}\right)$*If*A=B*then*$\lambda_{A}\left(\mu_{\tau }\right)=\lambda_{B}\left(\mu_{\tau }\right),\vartheta_{A}\left(\mu_{\tau }\right)=\vartheta_{B}\left(\mu_{\tau }\right)$*and*$d_{A}^{\prime }\left(\mu_{\tau }\right)=d_{B}^{\prime }\left(\mu_{\tau }\right)$*Therefore*, $\Delta_{1\;AB}\left(\mu_{\tau }\right)=0,\Delta_{2\;AB}\left(\mu_{\tau }\right)=0\;and\;\Delta_{3\;AB}\left(\mu_{\tau }\right)=0$*i*.*e*., $L_{m}\left(A,B\right)=1$.

*If*$L_{m}\left(A,B\right)=1$*then*

$$\Delta_{1\;AB}\left(\mu_{\tau }\right)=\frac{1}{m_{1}+1}│m_{1}\left(\lambda_{A}\left(\mu_{\tau }\right)-\lambda_{B}\left(\mu_{\tau }\right)\right)-\left(\vartheta_{A}\left(\mu_{\tau }\right)-\vartheta_{B}\left(\mu_{\tau }\right)\right)-\left(d_{A}^{\prime }\left(\mu_{\tau }\right)-d_{B}^{\prime }\left(\mu_{\tau }\right)\right)│=0$$

$$\Delta_{2\;AB}\left(\mu_{\tau }\right)=\frac{1}{2m_{2}+1}│m_{2}\left(\vartheta_{A}\left(\mu_{\tau }\right)-\vartheta_{B}\left(\mu_{\tau }\right)\right)-\left(\lambda_{A}\left(\mu_{\tau }\right)-\lambda_{B}\left(\mu_{\tau }\right)\right)+\left(d_{A}^{\prime }\left(\mu_{\tau }\right)-d_{B}^{\prime }\left(\mu_{\tau }\right)\right)│=0$$

$$\Delta_{3\;AB}\left(\mu_{\tau }\right)=\frac{1}{2m_{3}+1}│m_{3}\left(d_{A}^{\prime }\left(\mu_{\tau }\right)-d_{B}^{\prime }\left(\mu_{\tau }\right)\right)-\left(\lambda_{A}\left(\mu_{\tau }\right)-\lambda_{B}\left(\mu_{\tau }\right)\right)+\left(\vartheta_{A}\left(\mu_{\tau }\right)-\vartheta_{B}\left(\mu_{\tau }\right)\right)│=0$$

*By the definition of absolute value*, *we have*:

$$m_{1}\left(\lambda_{A}\left(\mu_{\tau }\right)-\lambda_{B}\left(\mu_{\tau }\right)\right)-\left(\vartheta_{A}\left(\mu_{\tau }\right)-\vartheta_{B}\left(\mu_{\tau }\right)\right)-\left(d_{A}^{\prime }\left(\mu_{\tau }\right)-d_{B}^{\prime }\left(\mu_{\tau }\right)\right)=0$$

$$m_{2}\left(\vartheta_{A}\left(\mu_{\tau }\right)-\vartheta_{B}\left(\mu_{\tau }\right)\right)-\left(\lambda_{A}\left(\mu_{\tau }\right)-\lambda_{B}\left(\mu_{\tau }\right)\right)+\left(d_{A}^{\prime }\left(\mu_{\tau }\right)-d_{B}^{\prime }\left(\mu_{\tau }\right)\right)=0$$

$$m_{3}\left(d_{A}^{\prime }\left(\mu_{\tau }\right)-d_{B}^{\prime }\left(\mu_{\tau }\right)\right)-\left(\lambda_{A}\left(\mu_{\tau }\right)-\lambda_{B}\left(\mu_{\tau }\right)\right)+\left(\vartheta_{A}\left(\mu_{\tau }\right)-\vartheta_{B}\left(\mu_{\tau }\right)\right)=0$$

$$i.e.\;\left(\begin{array}{ccc} m_{1} & -1 & -1\\ -1 & m_{2} & 1\\ -1 & 1 & m_{3} \end{array}\right)\;\left(\begin{array}{c} \lambda_{A}\left(\mu_{\tau }\right)-\lambda_{B}\left(\mu_{\tau }\right)\\ \vartheta_{A}\left(\mu_{\tau }\right)-\vartheta_{B}\left(\mu_{\tau }\right)\\ d_{A}^{\prime }\left(\mu_{\tau }\right)-d_{B}^{\prime }\left(\mu_{\tau }\right) \end{array}\right)=\left(\begin{array}{c} 0\\ 0\\ 0 \end{array}\right)$$

*Since*$\frac{1}{m_{1}+1}+\frac{1}{{2m}_{2}+1}+\frac{1}{{2m}_{3}+1}\in (0\text{,}1]$*then*$2\leq m_{1}m_{2}m_{3}-\left(m_{1}+m_{3}+m_{3}\right)$.

*By the definition of matrix determinant*, *we can get*:

$$\left|\begin{array}{ccc} m_{1} & -1 & -1\\ -1 & m_{2} & 1\\ -1 & 1 & m_{3} \end{array}\right|=m_{1}m_{2}m_{3}+2-\left(m_{1}+m_{3}+m_{3}\right)\geq 4$$

*Therefore*, *we have*

$$\left(\begin{array}{c} \lambda_{A}\left(\mu_{\tau }\right)-\lambda_{B}\left(\mu_{\tau }\right)\\ \vartheta_{A}\left(\mu_{\tau }\right)-\vartheta_{B}\left(\mu_{\tau }\right)\\ d_{A}^{\prime }\left(\mu_{\tau }\right)-d_{B}^{\prime }\left(\mu_{\tau }\right) \end{array}\right)={\left(\begin{array}{ccc} m_{1} & -1 & -1\\ -1 & m_{2} & 1\\ -1 & 1 & m_{3} \end{array}\right)}^{-1}\left(\begin{array}{c} 0\\ 0\\ 0 \end{array}\right)=\left(\begin{array}{c} 0\\ 0\\ 0 \end{array}\right)$$

It means that $\lambda_{A}\left(\mu_{\tau }\right)=\lambda_{B}\left(\mu_{\tau }\right),\vartheta_{A}\left(\mu_{\tau }\right)=\vartheta_{B}\left(\mu_{\tau }\right)$ and $d_{A}^{\prime }\left(\mu_{\tau }\right)=d_{B}^{\prime }\left(\mu_{\tau }\right)$ then A=B.

$\left(L_{3}\right)$*Based on the definition of absolute value*, *we can get the following equations*:

$$\Delta_{1\;AB}\left(\mu_{\tau }\right)=\frac{1}{m_{1}+1}│m_{1}\left(\lambda_{A}\left(\mu_{\tau }\right)-\lambda_{B}\left(\mu_{\tau }\right)\right)-\left(\vartheta_{A}\left(\mu_{\tau }\right)-\vartheta_{B}\left(\mu_{\tau }\right)\right)-\left(d_{A}^{\prime }\left(\mu_{\tau }\right)-d_{B}^{\prime }\left(\mu_{\tau }\right)\right)│$$

$$=\frac{1}{m_{1}+1}│\left(-1\right)\left[m_{1}\left(\lambda_{B}\left(\mu_{\tau }\right)-\lambda_{A}\left(\mu_{\tau }\right)\right)-\left(\vartheta_{B}\left(\mu_{\tau }\right)-\vartheta_{A}\left(\mu_{\tau }\right)\right)-\left(d_{B}^{\prime }\left(\mu_{\tau }\right)-d_{A}^{\prime }\left(\mu_{\tau }\right)\right)\right]│$$

$$=\frac{1}{m_{1}+1}│m_{1}\left(\lambda_{B}\left(\mu_{\tau }\right)-\lambda_{A}\left(\mu_{\tau }\right)\right)-\left(\vartheta_{B}\left(\mu_{\tau }\right)-\vartheta_{A}\left(\mu_{\tau }\right)\right)-\left(d_{B}^{\prime }\left(\mu_{\tau }\right)-d_{A}^{\prime }\left(\mu_{\tau }\right)\right)│$$

$${=\Delta }_{1\;BA\;}\left(\mu_{\tau }\right)$$

$$\Delta_{2\;AB}\left(\mu_{\tau }\right)=\frac{1}{2m_{2}+1}│m_{2}\left(\vartheta_{A}\left(\mu_{\tau }\right)-\vartheta_{B}\left(\mu_{\tau }\right)\right)-\left(\lambda_{A}\left(\mu_{\tau }\right)-\lambda_{B}\left(\mu_{\tau }\right)\right)+\left(d_{A}^{\prime }\left(\mu_{\tau }\right)-d_{B}^{\prime }\left(\mu_{\tau }\right)\right)│$$

$$=\frac{1}{2m_{2}+1}│\left(-1\right)\left[m_{2}\left(\vartheta_{B}\left(\mu_{\tau }\right)-\vartheta_{A}\left(\mu_{\tau }\right)\right)-\left(\lambda_{B}\left(\mu_{\tau }\right)-\lambda_{A}\left(\mu_{\tau }\right)\right)+\left(d_{B}^{\prime }\left(\mu_{\tau }\right)-d_{A}^{\prime }\left(\mu_{\tau }\right)\right)\right]│$$

$$=\frac{1}{2m_{2}+1}│m_{2}\left(\vartheta_{B}\left(\mu_{\tau }\right)-\vartheta_{A}\left(\mu_{\tau }\right)\right)-\left(\lambda_{B}\left(\mu_{\tau }\right)-\lambda_{A}\left(\mu_{\tau }\right)\right)+\left(d_{B}^{\prime }\left(\mu_{\tau }\right)-d_{A}^{\prime }\left(\mu_{\tau }\right)\right)│$$

$${=\Delta }_{2\;BA\;}\left(\mu_{\tau }\right)$$

$$\Delta_{3\;AB}\left(\mu_{\tau }\right)=\frac{1}{2m_{3}+1}│m_{3}\left(d_{A}^{\prime }\left(\mu_{\tau }\right)-d_{B}^{\prime }\left(\mu_{\tau }\right)\right)-\left(\lambda_{A}\left(\mu_{\tau }\right)-\lambda_{B}\left(\mu_{\tau }\right)\right)+\left(\vartheta_{A}\left(\mu_{\tau }\right)-\vartheta_{B}\left(\mu_{\tau }\right)\right)│$$

$$=\frac{1}{2m_{3}+1}│\left(-1\right)\left[│m_{3}\left(d_{B}^{\prime }\left(\mu_{\tau }\right)-d_{A}^{\prime }\left(\mu_{\tau }\right)\right)-\left(\lambda_{B}\left(\mu_{\tau }\right)-\lambda_{A}\left(\mu_{\tau }\right)\right)+\left(\vartheta_{B}\left(\mu_{\tau }\right)-\vartheta_{A}\left(\mu_{\tau }\right)\right)\right]│$$

$$=\frac{1}{2m_{3}+1}│m_{3}\left(d_{B}^{\prime }\left(\mu_{\tau }\right)-d_{A}^{\prime }\left(\mu_{\tau }\right)\right)-\left(\lambda_{B}\left(\mu_{\tau }\right)-\lambda_{A}\left(\mu_{\tau }\right)\right)+\left(\vartheta_{B}\left(\mu_{\tau }\right)-\vartheta_{A}\left(\mu_{\tau }\right)\right)│$$

$${=\Delta }_{3\;BA\;}\left(\mu_{\tau }\right)$$

$$L_{m}\left(A,B\right)=L_{m}\left(B,A\right)$$

$\left(L_{4}\right)$*Therefore*A⊆B⊆C*then*$\lambda_{A}\left(\mu_{\tau }\right)\leq \lambda_{B}\left(\mu_{\tau }\right)\leq \lambda_{C}\left(\mu_{\tau }\right)$ , $\vartheta_{C}\left(\mu_{\tau }\right)\leq \vartheta_{B}\left(\mu_{\tau }\right)\leq \vartheta_{A}\left(\mu_{\tau }\right)$, $d_{C}^{\prime }\left(\mu_{\tau }\right)\leq d_{B}^{\prime }\left(\mu_{\tau }\right)\leq d_{A}^{\prime }\left(\mu_{\tau }\right)$.

*Therefore*, *we can have*

$$m_{1}\left(\lambda_{A}\left(\mu_{\tau }\right)-\vartheta_{A}\left(\mu_{\tau }\right)-d_{A}^{\prime }\left(\mu_{\tau }\right)\right)\leq m_{1}\left(\lambda_{B}\left(\mu_{\tau }\right)-\vartheta_{B}\left(\mu_{\tau }\right)-d_{B}^{\prime }\left(\mu_{\tau }\right)\right)\leq m_{1}\left(\lambda_{C}\left(\mu_{\tau }\right)-\vartheta_{C}\left(\mu_{\tau }\right)-d_{C}^{\prime }\left(\mu_{\tau }\right)\right)$$

$$m_{2}\left(\vartheta_{C}\left(\mu_{\tau }\right)-\lambda_{C}\left(\mu_{\tau }\right)-d_{C}^{\prime }\left(\mu_{\tau }\right)\right)\leq m_{2}\left(\vartheta_{B}\left(\mu_{\tau }\right)-\lambda_{B}\left(\mu_{\tau }\right)-d_{B}^{\prime }\left(\mu_{\tau }\right)\right)\leq m_{2}\left(\vartheta_{A}\left(\mu_{\tau }\right)-\lambda_{A}\left(\mu_{\tau }\right)-d_{A}^{\prime }\left(\mu_{\tau }\right)\right)$$

$$m_{3}\left(d_{C}^{\prime }\left(\mu_{\tau }\right)-\lambda_{C}\left(\mu_{\tau }\right)-\vartheta_{C}\left(\mu_{\tau }\right)\right)\leq m_{3}\left(d_{B}^{\prime }\left(\mu_{\tau }\right)-\lambda_{B}\left(\mu_{\tau }\right)-\vartheta_{B}\left(\mu_{\tau }\right)\right)\leq m_{3}\left(d_{A}^{\prime }\left(\mu_{\tau }\right)-\lambda_{A}\left(\mu_{\tau }\right)-\vartheta_{A}\left(\mu_{\tau }\right)\right)$$

*By the property of inequality*, *we can obtain*:

$${│m}_{1}\left(\lambda_{A}\left(\mu_{\tau }\right)-\vartheta_{A}\left(\mu_{\tau }\right)-d_{A}^{\prime }\left(\mu_{\tau }\right)\right)-m_{1}\left(\lambda_{B}\left(\mu_{\tau }\right)-\vartheta_{B}\left(\mu_{\tau }\right)-d_{B}^{\prime }\left(\mu_{\tau }\right)\right)│$$

$${\leq │m}_{1}\left(\lambda_{A}\left(\mu_{\tau }\right)-\vartheta_{A}\left(\mu_{\tau }\right)-d_{A}^{\prime }\left(\mu_{\tau }\right)\right)-m_{1}\left(\lambda_{C}\left(\mu_{\tau }\right)-\vartheta_{C}\left(\mu_{\tau }\right)-d_{C}^{\prime }\left(\mu_{\tau }\right)\right)│$$

$$│m_{2}\left(\vartheta_{A}\left(\mu_{\tau }\right)-\lambda_{A}\left(\mu_{\tau }\right)-d_{A}^{\prime }\left(\mu_{\tau }\right)\right)-m_{2}\left(\vartheta_{B}\left(\mu_{\tau }\right)-\lambda_{B}\left(\mu_{\tau }\right)-d_{B}^{\prime }\left(\mu_{\tau }\right)\right)│$$

$${\leq │m}_{2}\left(\vartheta_{A}\left(\mu_{\tau }\right)-\lambda_{A}\left(\mu_{\tau }\right)-d_{A}^{\prime }\left(\mu_{\tau }\right)\right)-m_{2}\left(\vartheta_{C}\left(\mu_{\tau }\right)-\lambda_{C}\left(\mu_{\tau }\right)-d_{C}^{\prime }\left(\mu_{\tau }\right)\right)│$$

$${│m}_{3}\left(d_{A}^{\prime }\left(\mu_{\tau }\right)-\lambda_{A}\left(\mu_{\tau }\right)-\vartheta_{A}\left(\mu_{\tau }\right)\right)-m_{3}\left(d_{B}^{\prime }\left(\mu_{\tau }\right)-\lambda_{B}\left(\mu_{\tau }\right)-\vartheta_{B}\left(\mu_{\tau }\right)\right)│$$

$${\leq │m}_{3}\left(d_{A}^{\prime }\left(\mu_{\tau }\right)-\lambda_{A}\left(\mu_{\tau }\right)-\vartheta_{A}\left(\mu_{\tau }\right)\right)-m_{3}\left(d_{C}^{\prime }\left(\mu_{\tau }\right)-\lambda_{C}\left(\mu_{\tau }\right)-\vartheta_{C}\left(\mu_{\tau }\right)\right)│$$

$$\Delta_{2\;AB}\left(\mu_{\tau }\right)\leq \Delta_{1\;AC}\left(\mu_{\tau }\right),\Delta_{2\;AB}\left(\mu_{\tau }\right)\leq \Delta_{2\;AC}\left(\mu_{\tau }\right),\Delta_{3\;AB}\left(\mu_{\tau }\right)\leq \Delta_{3\;AC}\left(\mu_{\tau }\right)$$

*Therefore*, *we have*

$$1-{\left[\frac{1}{3n}\sum_{\tau =1}^{n}\Delta_{1\;AC}^{p}\left(\mu_{\tau }\right)+\Delta_{2\;AC}^{p}\left(\mu_{\tau }\right)+\Delta_{3\;AC}^{p}\left(\mu_{\tau }\right)\right]}^{\frac{1}{p}}=1-{\left[\frac{1}{3n}\sum_{\tau =1}^{n}\Delta_{1\;AB}^{p}\left(\mu_{\tau }\right)+\Delta_{2\;AB}^{p}\left(\mu_{\tau }\right)+\Delta_{3\;AB}^{p}\left(\mu_{\tau }\right)\right]}^{\frac{1}{p}}$$

*It means that*$L_{m}\left(A,C\right)\leq L_{m}\left(A,B\right)$.

*Similarity*, *we have*$L_{m}\left(A,C\right)\leq L_{m}\left(B,C\right)$.

(1)*When*$m_{1}=0,m_{2}=m_{3}=+\infty$, Eq. (14)*can be written as*:

$$L_{1}\left(A,B\right)=1-{\left[\frac{1}{3n}\sum_{\tau =1}^{n}\left(│\left(\vartheta_{A}\left(\mu_{\tau }\right)-\vartheta_{B}\left(\mu_{\tau }\right)\right)+\left({d^{\prime }}_{A}\left(\mu_{\tau }\right)-{d^{\prime }}_{B}\left(\mu_{\tau }\right)\right){│}^{P}+\frac{│\left(\vartheta_{A}\left(\mu_{\tau }\right)-\vartheta_{B}\left(\mu_{\tau }\right)\right){│}^{P}}{2^{P}}+\frac{│\left({d^{\prime }}_{A}\left(\mu_{\tau }\right)-{d^{\prime }}_{B}\left(\mu_{\tau }\right)\right){│}^{P}}{2^{P}}\right)\right]}^{\frac{1}{p}}$$

(2)*When*$m_{1}=m_{2}=+\infty m_{3}=0$, Eq. (14)*can be written as*:

$$L_{2}\left(A,B\right)=1-{\left[\frac{1}{3n}\sum_{\tau =1}^{n}\left(│\left(\lambda_{A}\left(\mu_{\tau }\right)-\lambda_{B}\left(\mu_{\tau }\right)\right){│}^{P}+\frac{│\left(\vartheta_{A}\left(\mu_{\tau }\right)-\vartheta_{B}\left(\mu_{\tau }\right)\right){│}^{P}}{2^{P}}+\frac{│\left(\left(\vartheta_{A}\left(\mu_{\tau }\right)-\vartheta_{B}\left(\mu_{\tau }\right)\right)-\left(\lambda_{A}\left(\mu_{\tau }\right)-\lambda_{B}\left(\mu_{\tau }\right)\right)\right){│}^{P}}{2^{P}}\right)\right]}^{\frac{1}{p}}$$

Theorem 2 *For any two SVNSs*$A=\left\{\left(\mu_{\tau },\lambda_{A}\left(\mu_{\tau }\right),\vartheta_{A}\left(\mu_{\tau }\right),d_{A}^{\prime }\left(\mu_{\tau }\right)\right)│\mu_{\tau }\in X\right\}$*and*$B=\left\{\left(\left(\mu_{\tau },\lambda_{B}\left(\mu_{\tau }\right),\vartheta_{B}\left(\mu_{\tau }\right),d_{B}^{\prime }\left(\mu_{\tau }\right)\right)\right)│\mu_{\tau }\in X\right\}$*on*$X=\left\{\mu_{1},\mu_{2},\ldots ,\mu_{n}\right\}$$\omega_{\tau }\in \left[0,1\right]$*and*$\sum_{\tau =1}^{n}\omega_{\tau }=1$*the function*$L_{\omega }:SVNSs\left(\mu \right)\times SVNSs\left(\mu \right)\to \left[0,3\right]$*defined by*

$$L_{\omega }\left(A,B\right)=1-{\left[\frac{1}{3n}\sum_{\tau =1}^{n}\omega_{\tau }\left(\Delta_{1\;AB}^{p}\left(\mu_{\tau }\right)+\Delta_{2\;AB}^{p}\left(\mu_{\tau }\right)+\Delta_{3\;AB}^{p}\left(\mu_{\tau }\right)\right)\right]}^{\frac{1}{p}}$$

$L_{m}\left(A,B\right)$*Is a similarity measure between*A*and*B, *where*,

$$\Delta_{1\;AB}\left(\mu_{\tau }\right)=\frac{1}{m_{1}+1}│m_{1}\left(\lambda_{A}\left(\mu_{\tau }\right)-\lambda_{B}\left(\mu_{\tau }\right)\right)-\left(\vartheta_{A}\left(\mu_{\tau }\right)-\vartheta_{B}\left(\mu_{\tau }\right)\right)-\left(d_{A}^{\prime }\left(\mu_{\tau }\right)-d_{B}^{\prime }\left(\mu_{\tau }\right)\right)│m_{1}\in [0,+\infty )\text{,}$$

$$\Delta_{2\;AB}\left(\mu_{\tau }\right)=\frac{1}{2m_{2}+1}│m_{2}\left(\vartheta_{A}\left(\mu_{\tau }\right)-\vartheta_{B}\left(\mu_{\tau }\right)\right)-\left(\lambda_{A}\left(\mu_{\tau }\right)-\lambda_{B}\left(\mu_{\tau }\right)\right)-\left(d_{A}^{\prime }\left(\mu_{\tau }\right)-d_{B}^{\prime }\left(\mu_{\tau }\right)\right)│m_{2}\in [0,+\infty )\text{,}$$

$$\Delta_{3\;AB}\left(\mu_{\tau }\right)=\frac{1}{2m_{3}+1}│m_{3}\left(d_{A}^{\prime }\left(\mu_{\tau }\right)-d_{B}^{\prime }\left(\mu_{\tau }\right)\right)-\left(\lambda_{A}\left(\mu_{\tau }\right)-\lambda_{B}\left(\mu_{\tau }\right)\right)-\left(\vartheta_{A}\left(\mu_{\tau }\right)-\vartheta_{B}\left(\mu_{\tau }\right)\right)│m_{3}\in [0,+\infty )\text{,}$$

$$\frac{1}{m_{1}+1}+\frac{1}{{2m}_{2}+1}+\frac{1}{{2m}_{3}+1}\in \left[0,3\right]$$

*and*p*is any positive integer*.

***Proof*:***In order to prove that* Eq. (18) *is a SMs*, *we only need to prove* Eq. (14) *satisfies axioms* $\left(L_{1}\right)-\left(L_{4}\right)$ $A=\left\{\left(\mu_{\tau },\lambda_{A}\left(\mu_{\tau }\right),\vartheta_{A}\left(\mu_{\tau }\right),d_{A}^{\prime }\left(\mu_{\tau }\right)\right)│\mu_{\tau }\in X\right\}$ *and* $B=\left\{\left(\left(\mu_{\tau },\lambda_{B}\left(\mu_{\tau }\right),\vartheta_{B}\left(\mu_{\tau }\right),d_{B}^{\prime }\left(\mu_{\tau }\right)\right)\right)│\mu_{\tau }\in X\right\}$ *and* $C=\left\{\left(\left(\mu_{\tau },\lambda_{C}\left(\mu_{\tau }\right),\vartheta_{C}\left(\mu_{\tau }\right),d_{C}^{\prime }\left(\mu_{\tau }\right)\right)\right)│\mu_{\tau }\in X\right\}$ *be any three SVNSs on* $X=\left\{\mu_{1},\mu_{2},\ldots ,\mu_{n}\right\}$.

$\left(L_{1}\right)$*We can write the following equations*:

$$\Delta_{1\;AB}\left(\mu_{\tau }\right)=\frac{1}{m_{1}+1}│m_{1}\left(\lambda_{A}\left(\mu_{\tau }\right)-\lambda_{B}\left(\mu_{\tau }\right)\right)-\left(\vartheta_{A}\left(\mu_{\tau }\right)-\vartheta_{B}\left(\mu_{\tau }\right)\right)-\left(d_{A}^{\prime }\left(\mu_{\tau }\right)-d_{B}^{\prime }\left(\mu_{\tau }\right)\right)│m_{1}\in [0,+\infty )$$

$$=\frac{1}{m_{1}+1}│\left(m_{1}\lambda_{A}\left(\mu_{\tau }\right)-\vartheta_{A}\left(\mu_{\tau }\right)-d_{A}^{\prime }\left(\mu_{\tau }\right)\right)-\left(m_{1}\lambda_{B}\left(\mu_{\tau }\right)-\vartheta_{B}\left(\mu_{\tau }\right)-d_{B}^{\prime }\left(\mu_{\tau }\right)\right)│$$

$$\Delta_{2\;AB}\left(\mu_{\tau }\right)=\frac{1}{2m_{2}+1}│m_{2}\left(\vartheta_{A}\left(\mu_{\tau }\right)-\vartheta_{B}\left(\mu_{\tau }\right)\right)-\left(\lambda_{A}\left(\mu_{\tau }\right)-\lambda_{B}\left(\mu_{\tau }\right)\right)+\left(d_{A}^{\prime }\left(\mu_{\tau }\right)-d_{B}^{\prime }\left(\mu_{\tau }\right)\right)│m_{2}\in [0,+\infty )$$

$$=\frac{1}{2m_{2}+1}│\left(m_{2}\vartheta_{A}\left(\mu_{\tau }\right)-\lambda_{A}\left(\mu_{\tau }\right)-d_{A}^{\prime }\left(\mu_{\tau }\right)\right)-\left(m_{2}\vartheta_{B}\left(\mu_{\tau }\right)-\lambda_{B}\left(\mu_{\tau }\right)-d_{B}^{\prime }\left(\mu_{\tau }\right)\right)│$$

$$\Delta_{3\;AB}\left(\mu_{\tau }\right)=\frac{1}{2m_{3}+1}│m_{3}\left(d_{A}^{\prime }\left(\mu_{\tau }\right)-d_{B}^{\prime }\left(\mu_{\tau }\right)\right)-\left(\lambda_{A}^{n}\left(\mu_{\tau }\right)-\lambda_{B}\left(\mu_{\tau }\right)\right)+\left(\vartheta_{A}\left(\mu_{\tau }\right)-\vartheta_{B}\left(\mu_{\tau }\right)\right)│m_{3}\in [0,+\infty )$$

$$=\frac{1}{2m_{3}+1}│\left({{m_{3}d}^{\prime }}_{A}\left(\mu_{\tau }\right)-\lambda_{A}\left(\mu_{\tau }\right)-\vartheta_{A}\left(\mu_{\tau }\right)\right)-\left(m_{3}d_{B}^{\prime }\left(\mu_{\tau }\right)-\lambda_{B}\left(\mu_{\tau }\right)-\vartheta_{B}\left(\mu_{\tau }\right)\right)│$$

*By*$\lambda_{A}\left(\mu_{\tau }\right),\vartheta_{A}\left(\mu_{\tau }\right),d_{A}^{\prime }\left(\mu_{\tau }\right),\lambda_{B}\left(\mu_{\tau }\right),\vartheta_{B}\left(\mu_{\tau }\right),d_{B}^{\prime }\left(\mu_{\tau }\right)\in \left[0,1\right]$*and*$\lambda_{A}\left(\mu_{\tau }\right)+\vartheta_{A}\left(\mu_{\tau }\right)+d_{A}^{\prime }\left(\mu_{\tau }\right)\leq 1\;\lambda_{B}\left(\mu_{\tau }\right)+\vartheta_{B}\left(\mu_{\tau }\right)+d_{B}^{\prime }\left(\mu_{\tau }\right)\leq 3$.

*We have*

$$-1\leq m_{1}\lambda_{A}\left(\mu_{\tau }\right)-\vartheta_{A}\left(\mu_{\tau }\right)-d_{A}^{\prime }\left(\mu_{\tau }\right)\leq m_{1}-m_{1}\leq -\left({m_{1}\lambda }_{B}\left(\mu_{\tau }\right)-\vartheta_{B}\left(\mu_{\tau }\right)-d_{B}^{\prime }\left(\mu_{\tau }\right)\right)\leq 1$$

$$0\leq │\left(m_{1}\lambda_{A}\left(\mu_{\tau }\right)-\vartheta_{A}\left(\mu_{\tau }\right)-d_{A}^{\prime }\left(\mu_{\tau }\right)\right)-\left(m_{1}\lambda_{B}\left(\mu_{\tau }\right)-\vartheta_{B}\left(\mu_{\tau }\right)-d_{B}^{\prime }\left(\mu_{\tau }\right)\right)│\leq m_{1}+1\;i.e,{0\leq \Delta }_{1\;AB}\left(\mu_{\tau }\right)\leq 1$$

*Then*

$$-1\leq m_{2}\vartheta_{A}\left(\mu_{\tau }\right)-\lambda_{A}\left(\mu_{\tau }\right)+d_{A}^{\prime }\left(\mu_{\tau }\right)\leq 1⋁m_{2}-\left(1⋁m_{2}\right)\leq -\left({m_{2}\vartheta }_{B}\left(\mu_{\tau }\right)-\lambda_{B}\left(\mu_{\tau }\right)+d_{B}^{\prime }\left(\mu_{\tau }\right)\right)\leq 1$$

*Similarly*, *we get the following inequalities*:

$$-1\leq m_{3}d_{A}^{\prime }\left(\mu_{\tau }\right)-\lambda_{A}\left(\mu_{\tau }\right)+\vartheta_{A}\left(\mu_{\tau }\right)\leq 1⋁m_{3}-\left(1⋁m_{3}\right)\leq -\left(m_{3}d_{B}^{\prime }\left(\mu_{\tau }\right)-\lambda_{B}\left(\mu_{\tau }\right)+\vartheta_{B}\left(\mu_{\tau }\right)\right)\leq 1$$

*Then we obtain*:

$$0\leq │\left(m_{2}\vartheta_{A}\left(\mu_{\tau }\right)-\lambda_{A}\left(\mu_{\tau }\right)+d_{A}^{\prime }\left(\mu_{\tau }\right)\right)-\left({m_{2}\vartheta }_{B}\left(\mu_{\tau }\right)-\lambda_{B}\left(\mu_{\tau }\right)+d_{B}^{\prime }\left(\mu_{\tau }\right)\right)│\leq 2⋁m_{2}$$

$$0\leq │\left(m_{3}d_{A}^{\prime }\left(\mu_{\tau }\right)-\lambda_{A}\left(\mu_{\tau }\right)+\vartheta_{A}\left(\mu_{\tau }\right)\right)-\left(m_{3}d_{B}^{\prime }\left(\mu_{\tau }\right)-\lambda_{B}\left(\mu_{\tau }\right)+\vartheta_{B}\left(\mu_{\tau }\right)\right)│\leq 2⋁m_{3}$$

*It means that*:

$$0\leq \Delta_{2\;AB}\left(\mu_{\tau }\right)=\frac{1}{2m_{2}+1}│\left(m_{2}\vartheta_{A}\left(\mu_{\tau }\right)-\lambda_{A}\left(\mu_{\tau }\right)+d_{A}^{\prime }\left(\mu_{\tau }\right)\right)-\left({m_{2}\vartheta }_{B}\left(\mu_{\tau }\right)-\lambda_{B}\left(\mu_{\tau }\right)+d_{B}^{\prime }\left(\mu_{\tau }\right)\right)│\leq \frac{1}{m_{2}+1}⋁\frac{1}{2}\leq 1$$

$$0\leq \Delta_{3\;AB}\left(\mu_{\tau }\right)=\frac{1}{2m_{3}+1}│\left(m_{3}d_{A}^{\prime }\left(\mu_{\tau }\right)-\lambda_{A}\left(\mu_{\tau }\right)+\vartheta_{A}\left(\mu_{\tau }\right)\right)-\left(m_{3}d_{B}^{\prime }\left(\mu_{\tau }\right)-\lambda_{B}\left(\mu_{\tau }\right)+\vartheta_{B}\left(\mu_{\tau }\right)\right)│\leq \frac{1}{m_{3}+1}⋁\frac{1}{2}\leq 1$$

*Finally*, *we have*:

$$0\leq 1-{\left[\frac{1}{3n}\sum_{\tau =1}^{n}\omega_{\tau }\left(\Delta_{1\;AB}^{p}\left(\mu_{\tau }\right)+\Delta_{2\;AB}^{p}\left(\mu_{\tau }\right)+\Delta_{3\;AB}^{p}\left(\mu_{\tau }\right)\right)\right]}^{\frac{1}{p}}\leq 1$$

*Therefore*,

$$\left(L_{1}\right)\;0\leq L_{m}\left(A,B\right)\leq 1$$

$\left(L_{2}\right)$*If*A=B*then*$\lambda_{A}\left(\mu_{\tau }\right)=\lambda_{B}\left(\mu_{\tau }\right),\vartheta_{A}\left(\mu_{\tau }\right)=\vartheta_{B}\left(\mu_{\tau }\right)$ and $d_{A}^{\prime }\left(\mu_{\tau }\right)=d_{B}^{\prime }\left(\mu_{\tau }\right)$ *Therefore*, $\Delta_{1\;AB}\left(\mu_{\tau }\right)=0,\Delta_{2\;AB}\left(\mu_{\tau }\right)=0\;and\;\Delta_{3\;AB}\left(\mu_{\tau }\right)=0$ *i*.*e*., $L_{m}\left(A,B\right)=1$.

*If*$L_{m}\left(A,B\right)=1$*then*

$$\Delta_{1\;AB}\left(\mu_{\tau }\right)=\frac{1}{m_{1}+1}│m_{1}\left(\lambda_{A}\left(\mu_{\tau }\right)-\lambda_{B}\left(\mu_{\tau }\right)\right)-\left(\vartheta_{A}\left(\mu_{\tau }\right)-\vartheta_{B}\left(\mu_{\tau }\right)\right)-\left(d_{A}^{\prime }\left(\mu_{\tau }\right)-d_{B}^{\prime }\left(\mu_{\tau }\right)\right)│=0$$

$$\Delta_{2\;AB}\left(\mu_{\tau }\right)=\frac{1}{2m_{2}+1}│m_{2}\left(\vartheta_{A}\left(\mu_{\tau }\right)-\vartheta_{B}\left(\mu_{\tau }\right)\right)-\left(\lambda_{A}\left(\mu_{\tau }\right)-\lambda_{B}\left(\mu_{\tau }\right)\right)+\left(d_{A}^{\prime }\left(\mu_{\tau }\right)-d_{B}^{\prime }\left(\mu_{\tau }\right)\right)│=0$$

$$\Delta_{3\;AB}\left(\mu_{\tau }\right)=\frac{1}{2m_{3}+1}│m_{3}\left(d_{A}^{\prime }\left(\mu_{\tau }\right)-d_{B}^{\prime }\left(\mu_{\tau }\right)\right)-\left(\lambda_{A}\left(\mu_{\tau }\right)-\lambda_{B}\left(\mu_{\tau }\right)\right)+\left(\vartheta_{A}\left(\mu_{\tau }\right)-\vartheta_{B}\left(\mu_{\tau }\right)\right)│=0$$

*By the definition of absolute value*, *we have*:

$$m_{1}\left(\lambda_{A}\left(\mu_{\tau }\right)-\lambda_{B}\left(\mu_{\tau }\right)\right)-\left(\vartheta_{A}\left(\mu_{\tau }\right)-\vartheta_{B}\left(\mu_{\tau }\right)\right)-\left(d_{A}^{\prime }\left(\mu_{\tau }\right)-d_{B}^{\prime }\left(\mu_{\tau }\right)\right)=0$$

$$m_{2}\left(\vartheta_{A}\left(\mu_{\tau }\right)-\vartheta_{B}\left(\mu_{\tau }\right)\right)-\left(\lambda_{A}\left(\mu_{\tau }\right)-\lambda_{B}\left(\mu_{\tau }\right)\right)+\left(d_{A}^{\prime }\left(\mu_{\tau }\right)-d_{B}^{\prime }\left(\mu_{\tau }\right)\right)=0$$

$$m_{3}\left(d_{A}^{\prime }\left(\mu_{\tau }\right)-d_{B}^{\prime }\left(\mu_{\tau }\right)\right)-\left(\lambda_{A}\left(\mu_{\tau }\right)-\lambda_{B}\left(\mu_{\tau }\right)\right)+\left(\vartheta_{A}\left(\mu_{\tau }\right)-\vartheta_{B}\left(\mu_{\tau }\right)\right)=0$$

$$i.e.\;\left(\begin{array}{ccc} m_{1} & -1 & -1\\ -1 & m_{2} & 1\\ -1 & 1 & m_{3} \end{array}\right)\;\left(\begin{array}{c} \lambda_{A}\left(\mu_{\tau }\right)-\lambda_{B}\left(\mu_{\tau }\right)\\ \vartheta_{A}\left(\mu_{\tau }\right)-\vartheta_{B}\left(\mu_{\tau }\right)\\ d_{A}^{\prime }\left(\mu_{\tau }\right)-d_{B}^{\prime }\left(\mu_{\tau }\right) \end{array}\right)=\left(\begin{array}{c} 0\\ 0\\ 0 \end{array}\right)$$

*Since*$\frac{1}{m_{1}+1}+\frac{1}{{2m}_{2}+1}+\frac{1}{{2m}_{3}+1}\in (0\text{,}1]$*then*$2\leq m_{1}m_{2}m_{3}-\left(m_{1}+m_{3}+m_{3}\right)$.

*By the definition of matrix determinant*, *we can get*:

$$\left|\begin{array}{ccc} m_{1} & -1 & -1\\ -1 & m_{2} & 1\\ -1 & 1 & m_{3} \end{array}\right|=m_{1}m_{2}m_{3}+2-\left(m_{1}+m_{3}+m_{3}\right)\geq 4$$

*Therefore*, *we have*

$$\left(\begin{array}{c} \lambda_{A}\left(\mu_{\tau }\right)-\lambda_{B}\left(\mu_{\tau }\right)\\ \vartheta_{A}\left(\mu_{\tau }\right)-\vartheta_{B}\left(\mu_{\tau }\right)\\ d_{A}^{\prime }\left(\mu_{\tau }\right)-d_{B}^{\prime }\left(\mu_{\tau }\right) \end{array}\right)={\left(\begin{array}{ccc} m_{1} & -1 & -1\\ -1 & m_{2} & 1\\ -1 & 1 & m_{3} \end{array}\right)}^{-1}\left(\begin{array}{c} 0\\ 0\\ 0 \end{array}\right)=\left(\begin{array}{c} 0\\ 0\\ 0 \end{array}\right)$$

It means that $\lambda_{A}\left(\mu_{\tau }\right)=\lambda_{B}\left(\mu_{\tau }\right),\vartheta_{A}\left(\mu_{\tau }\right)=\vartheta_{B}\left(\mu_{\tau }\right)$ and $d_{A}^{\prime }\left(\mu_{\tau }\right)=d_{B}^{\prime }\left(\mu_{\tau }\right)$ then A=B.

$\left(L_{3}\right)$*Based on the definition of absolute value*, *we can get the following equations*:

$$\Delta_{1\;AB}\left(\mu_{\tau }\right)=\frac{1}{m_{1}+1}│m_{1}\left(\lambda_{A}\left(\mu_{\tau }\right)-\lambda_{B}\left(\mu_{\tau }\right)\right)-\left(\vartheta_{A}\left(\mu_{\tau }\right)-\vartheta_{B}\left(\mu_{\tau }\right)\right)-\left(d_{A}^{\prime }\left(\mu_{\tau }\right)-d_{B}^{\prime }\left(\mu_{\tau }\right)\right)│$$

$$=\frac{1}{m_{1}+1}│\left(-1\right)\left[m_{1}\left(\lambda_{B}\left(\mu_{\tau }\right)-\lambda_{A}\left(\mu_{\tau }\right)\right)-\left(\vartheta_{B}\left(\mu_{\tau }\right)-\vartheta_{A}\left(\mu_{\tau }\right)\right)-\left(d_{B}^{\prime }\left(\mu_{\tau }\right)-d_{A}^{\prime }\left(\mu_{\tau }\right)\right)\right]│$$

$$=\frac{1}{m_{1}+1}│m_{1}\left(\lambda_{B}\left(\mu_{\tau }\right)-\lambda_{A}\left(\mu_{\tau }\right)\right)-\left(\vartheta_{B}\left(\mu_{\tau }\right)-\vartheta_{A}\left(\mu_{\tau }\right)\right)-\left(d_{B}^{\prime }\left(\mu_{\tau }\right)-d_{A}^{\prime }\left(\mu_{\tau }\right)\right)│$$

$${=\Delta }_{1\;BA\;}\left(\mu_{\tau }\right)$$

$$\Delta_{2\;AB}\left(\mu_{\tau }\right)=\frac{1}{2m_{2}+1}│m_{2}\left(\vartheta_{A}\left(\mu_{\tau }\right)-\vartheta_{B}\left(\mu_{\tau }\right)\right)-\left(\lambda_{A}\left(\mu_{\tau }\right)-\lambda_{B}\left(\mu_{\tau }\right)\right)+\left(d_{A}^{\prime }\left(\mu_{\tau }\right)-d_{B}^{\prime }\left(\mu_{\tau }\right)\right)│$$

$$=\frac{1}{2m_{2}+1}│\left(-1\right)\left[m_{2}\left(\vartheta_{B}\left(\mu_{\tau }\right)-\vartheta_{A}\left(\mu_{\tau }\right)\right)-\left(\lambda_{B}\left(\mu_{\tau }\right)-\lambda_{A}\left(\mu_{\tau }\right)\right)+\left(d_{B}^{\prime }\left(\mu_{\tau }\right)-d_{A}^{\prime }\left(\mu_{\tau }\right)\right)\right]│$$

$$=\frac{1}{2m_{2}+1}│m_{2}\left(\vartheta_{B}\left(\mu_{\tau }\right)-\vartheta_{A}\left(\mu_{\tau }\right)\right)-\left(\lambda_{B}\left(\mu_{\tau }\right)-\lambda_{A}\left(\mu_{\tau }\right)\right)+\left(d_{B}^{\prime }\left(\mu_{\tau }\right)-d_{A}^{\prime }\left(\mu_{\tau }\right)\right)│$$

$${=\Delta }_{2\;BA\;}\left(\mu_{\tau }\right)$$

$$\Delta_{3\;AB}\left(\mu_{\tau }\right)=\frac{1}{2m_{3}+1}│m_{3}\left(d_{A}^{\prime }\left(\mu_{\tau }\right)-d_{B}^{\prime }\left(\mu_{\tau }\right)\right)-\left(\lambda_{A}\left(\mu_{\tau }\right)-\lambda_{B}\left(\mu_{\tau }\right)\right)+\left(\vartheta_{A}\left(\mu_{\tau }\right)-\vartheta_{B}\left(\mu_{\tau }\right)\right)│$$

$$=\frac{1}{2m_{3}+1}│\left(-1\right)\left[│m_{3}\left(d_{B}^{\prime }\left(\mu_{\tau }\right)-d_{A}^{\prime }\left(\mu_{\tau }\right)\right)-\left(\lambda_{B}\left(\mu_{\tau }\right)-\lambda_{A}\left(\mu_{\tau }\right)\right)+\left(\vartheta_{B}\left(\mu_{\tau }\right)-\vartheta_{A}\left(\mu_{\tau }\right)\right)\right]│$$

$$=\frac{1}{2m_{3}+1}│m_{3}\left(d_{B}^{\prime }\left(\mu_{\tau }\right)-d_{A}^{\prime }\left(\mu_{\tau }\right)\right)-\left(\lambda_{B}\left(\mu_{\tau }\right)-\lambda_{A}\left(\mu_{\tau }\right)\right)+\left(\vartheta_{B}\left(\mu_{\tau }\right)-\vartheta_{A}\left(\mu_{\tau }\right)\right)│$$

$${=\Delta }_{3\;BA\;}\left(\mu_{\tau }\right)$$

$$L_{m}\left(A,B\right)=L_{m}\left(B,A\right)$$

$\left(L_{4}\right)$*Therefore*A⊆B⊆C*then*$\lambda_{A}\left(\mu_{\tau }\right)\leq \lambda_{B}\left(\mu_{\tau }\right)\leq \lambda_{C}\left(\mu_{\tau }\right)$ , $\vartheta_{C}\left(\mu_{\tau }\right)\leq \vartheta_{B}\left(\mu_{\tau }\right)\leq \vartheta_{A}\left(\mu_{\tau }\right)$, $d_{C}^{\prime }\left(\mu_{\tau }\right)\leq d_{B}^{\prime }\left(\mu_{\tau }\right)\leq d_{A}^{\prime }\left(\mu_{\tau }\right)$.

*Therefore*, *we can have*

$$m_{1}\left(\lambda_{A}\left(\mu_{\tau }\right)-\vartheta_{A}\left(\mu_{\tau }\right)-d_{A}^{\prime }\left(\mu_{\tau }\right)\right)\leq m_{1}\left(\lambda_{B}\left(\mu_{\tau }\right)-\vartheta_{B}\left(\mu_{\tau }\right)-d_{B}^{\prime }\left(\mu_{\tau }\right)\right)\leq m_{1}\left(\lambda_{C}\left(\mu_{\tau }\right)-\vartheta_{C}\left(\mu_{\tau }\right)-d_{C}^{\prime }\left(\mu_{\tau }\right)\right)$$

$$m_{2}\left(\vartheta_{C}\left(\mu_{\tau }\right)-\lambda_{C}\left(\mu_{\tau }\right)-d_{C}^{\prime }\left(\mu_{\tau }\right)\right)\leq m_{2}\left(\vartheta_{B}\left(\mu_{\tau }\right)-\lambda_{B}\left(\mu_{\tau }\right)-d_{B}^{\prime }\left(\mu_{\tau }\right)\right)\leq m_{2}\left(\vartheta_{A}\left(\mu_{\tau }\right)-\lambda_{A}\left(\mu_{\tau }\right)-d_{A}^{\prime }\left(\mu_{\tau }\right)\right)$$

$$m_{3}\left(d_{C}^{\prime }\left(\mu_{\tau }\right)-\lambda_{C}\left(\mu_{\tau }\right)-\vartheta_{C}\left(\mu_{\tau }\right)\right)\leq m_{3}\left(d_{B}^{\prime }\left(\mu_{\tau }\right)-\lambda_{B}\left(\mu_{\tau }\right)-\vartheta_{B}\left(\mu_{\tau }\right)\right)\leq m_{3}\left(d_{A}^{\prime }\left(\mu_{\tau }\right)-\lambda_{A}\left(\mu_{\tau }\right)-\vartheta_{A}\left(\mu_{\tau }\right)\right)$$

*By the property of inequality*, *we can obtain*:

$${│m}_{1}\left(\lambda_{A}\left(\mu_{\tau }\right)-\vartheta_{A}\left(\mu_{\tau }\right)-d_{A}^{\prime }\left(\mu_{\tau }\right)\right)-m_{1}\left(\lambda_{B}\left(\mu_{\tau }\right)-\vartheta_{B}\left(\mu_{\tau }\right)-d_{B}^{\prime }\left(\mu_{\tau }\right)\right)│$$

$${\leq │m}_{1}\left(\lambda_{A}\left(\mu_{\tau }\right)-\vartheta_{A}\left(\mu_{\tau }\right)-d_{A}^{\prime }\left(\mu_{\tau }\right)\right)-m_{1}\left(\lambda_{C}\left(\mu_{\tau }\right)-\vartheta_{C}\left(\mu_{\tau }\right)-d_{C}^{\prime }\left(\mu_{\tau }\right)\right)│$$

$$│m_{2}\left(\vartheta_{A}\left(\mu_{\tau }\right)-\lambda_{A}\left(\mu_{\tau }\right)-d_{A}^{\prime }\left(\mu_{\tau }\right)\right)-m_{2}\left(\vartheta_{B}\left(\mu_{\tau }\right)-\lambda_{B}\left(\mu_{\tau }\right)-d_{B}^{\prime }\left(\mu_{\tau }\right)\right)│$$

$${\leq │m}_{2}\left(\vartheta_{A}\left(\mu_{\tau }\right)-\lambda_{A}\left(\mu_{\tau }\right)-d_{A}^{\prime }\left(\mu_{\tau }\right)\right)-m_{2}\left(\vartheta_{C}\left(\mu_{\tau }\right)-\lambda_{C}\left(\mu_{\tau }\right)-d_{C}^{\prime }\left(\mu_{\tau }\right)\right)│$$

$${│m}_{3}\left(d_{A}^{\prime }\left(\mu_{\tau }\right)-\lambda_{A}\left(\mu_{\tau }\right)-\vartheta_{A}\left(\mu_{\tau }\right)\right)-m_{3}\left(d_{B}^{\prime }\left(\mu_{\tau }\right)-\lambda_{B}\left(\mu_{\tau }\right)-\vartheta_{B}\left(\mu_{\tau }\right)\right)│$$

$${\leq │m}_{3}\left(d_{A}^{\prime }\left(\mu_{\tau }\right)-\lambda_{A}\left(\mu_{\tau }\right)-\vartheta_{A}\left(\mu_{\tau }\right)\right)-m_{3}\left(d_{C}^{\prime }\left(\mu_{\tau }\right)-\lambda_{C}\left(\mu_{\tau }\right)-\vartheta_{C}\left(\mu_{\tau }\right)\right)│$$

$$\Delta_{2\;AB}\left(\mu_{\tau }\right)\leq \Delta_{1\;AC}\left(\mu_{\tau }\right),\Delta_{2\;AB}\left(\mu_{\tau }\right)\leq \Delta_{2\;AC}\left(\mu_{\tau }\right),\Delta_{3\;AB}\left(\mu_{\tau }\right)\leq \Delta_{3\;AC}\left(\mu_{\tau }\right)$$

*Therefore*, *we have*

$$1-{\left[\frac{1}{3n}\sum_{\tau =1}^{n}\omega_{\tau }\left(\Delta_{1\;AC}^{p}\left(\mu_{\tau }\right)+\Delta_{2\;AC}^{p}\left(\mu_{\tau }\right)+\Delta_{3\;AC}^{p}\left(\mu_{\tau }\right)\right)\right]}^{\frac{1}{p}}=1-{\left[\frac{1}{3n}\sum_{\tau =1}^{n}\omega_{\tau }\left(\Delta_{1\;AB}^{p}\left(\mu_{\tau }\right)+\Delta_{2\;AB}^{p}\left(\mu_{\tau }\right)+\Delta_{3\;AB}^{p}\left(\mu_{\tau }\right)\right)\right]}^{\frac{1}{p}}$$

*It means that*$L_{m}\left(A,C\right)\leq L_{m}\left(A,B\right)$.

Similarity, we have $L_{m}\left(A,C\right)\leq L_{m}\left(B,C\right)$.

(1)*When*$m_{1}=0,m_{2}=m_{3}=+\infty$, Eq. (14)*can be written as*:

$$L_{1}\left(A,B\right)=1-{\left[\frac{1}{3n}\sum_{\tau =1}^{n}\omega_{\tau }\left(│\left(\vartheta_{A}\left(\mu_{\tau }\right)-\vartheta_{B}\left(\mu_{\tau }\right)\right)+\left({d^{\prime }}_{A}\left(\mu_{\tau }\right)-{d^{\prime }}_{B}\left(\mu_{\tau }\right)\right){│}^{P}+\frac{│\left(\vartheta_{A}\left(\mu_{\tau }\right)-\vartheta_{B}\left(\mu_{\tau }\right)\right){│}^{P}}{2^{P}}+\frac{│\left({d^{\prime }}_{A}\left(\mu_{\tau }\right)-{d^{\prime }}_{B}\left(\mu_{\tau }\right)\right){│}^{P}}{2^{P}}\right)\right]}^{\frac{1}{p}}$$

(2)*When*$m_{1}=m_{2}=+\infty m_{3}=0$, Eq. (14)*can be written as*:

(18) $$L_{2}\left(A,B\right)=1-{\left[\frac{1}{3n}\sum_{\tau =1}^{n}\omega_{\tau }\left(│\left(\lambda_{A}\left(\mu_{\tau }\right)-\lambda_{B}\left(\mu_{\tau }\right)\right){│}^{P}+\frac{│\left(\vartheta_{A}\left(\mu_{\tau }\right)-\vartheta_{B}\left(\mu_{\tau }\right)\right){│}^{P}}{2^{P}}+\frac{│\left(\left(\vartheta_{A}\left(\mu_{\tau }\right)-\vartheta_{B}\left(\mu_{\tau }\right)\right)-\left(\lambda_{A}\left(\mu_{\tau }\right)-\lambda_{B}\left(\mu_{\tau }\right)\right)\right){│}^{P}}{2^{P}}\right)\right]}^{\frac{1}{p}}$$

**Example 1:** Let A=(μ,0.99,0.78,0.22), B=(μ,0.99,0.66,0.99) and C=(μ,0.73,0.86,0.99) are three different SVNSs on X={μ}. A Is more similar to B than C to say L(A,B)>L(A,C). To prove the accuracy of this view for our proposed SMs $L_{m}$ and the current ones to be specific ${\;L}_{1}$, $L_{2}$, $L_{3}$, $L_{4},L_{5},L_{6}L_{7},L_{9},L_{10}$ $L_{11}$ $L_{12},L_{13},L_{m}$. We can see the obtained values of the SMs in Table 2.

**Table 2:** The result of example.

	L(φ,B)	L(φ,C)	**Relation**
$L_{1}$	0.7033	0.6300	L(A,B)>L(A,C)
$L_{2}$	0.8988	0.8889	L(A,B)>L(A,C)
$L_{3}$	1.000	1.000	L(A,B)=L(A,C)
$L_{4}$	0.3535	0.3535	L(A,B)=L(A,C)
$L_{5}$	0.8934	0.8358	L(A,B)>L(A,C)
$L_{6}$	0.3089	0.3089	L(A,B)=L(A,C)
$L_{7}$	0.7627	0.7009	L(A,B)>L(A,C)
$L_{8}$	0.3778	0.3778	L(A,B)=L(A,C)
$L_{9}$	0.9589	0.9589	L(A,B)=L(A,C)
$L_{10}$	0.2300	0.2300	L(A,B)=L(A,C)
$L_{11}$	0.8649	0.8391	L(A,B)>L(A,C)
$L_{12}$	0.8735	0.8740	L(A,B)<L(A,C)
$L_{13}$	0.7753	0.7762	L(A,B)<L(A,C)
$L_{m}$	0.6552	0.4113	L(A,B)>L(A,C)

*In this example show that the*$L_{1},L_{2},L_{5},L_{7},L_{11},L_{14}and{\;L}_{m}$*can be provide the best result of SMs***.**

## 5 Application of the proposed similarity measures

This section applies the suggested SMs to MADM problems, demonstrating that the expected SMs makes sense and is consistent with human cognition.

Let $X=\left\{\mu_{1},\mu_{2},\ldots \mu_{m}\right\}$ a set of attributes, the n alternatives $A_{\tau }=\left(A_{\tau j}\right)=\left\{\left(\mu_{j},\lambda_{A\tau }\left(\mu_{j}\right),\vartheta_{A\tau }\left(\mu_{j}\right),d_{A\tau }^{\prime }\left(\mu_{j}\right)\right)\right\}│\mu_{j}\in X$ Where $\mu_{j},\lambda_{A\tau }\left(\mu_{j}\right),\vartheta_{A\tau }\left(\mu_{j}\right),d_{A\tau }^{\prime }\left(\mu_{j}\right)\in \left[0,1\right]$ and $\lambda_{A\tau }\left(\mu_{j}\right)+\vartheta_{A\tau }\left(\mu_{j}\right)+d_{A\tau }^{\prime }\left(\mu_{j}\right)\leq 3$, $\lambda_{A\tau }\left(\mu_{\tau }\right)$ is a positive degree which is use to alternative $A_{\tau }$ satisfies the $\mu_{j}\left(\tau =\left\{1,2,\ldots n\right\},j=\left\{1,2,\ldots m\right\}\right)$. $\vartheta_{A\tau }\left(\mu_{\tau }\right)$ a neutral degree which is use to alternative $A_{\tau }$ does not satisfies the $\mu_{j}$. $d_{A\tau }^{\prime }\left(\mu_{\tau }\right)$ , negative degree which is use to alternative $A_{\tau }$ does not satisfies the ${\;\mu }_{j}$. The decision making is used to choose best alternative steps are following.

**Step 1:** Normalize decision alternatives.

In this process multi attribute decision making can be divided in to type's amount type and interest type. The amount type can be changed into interest type by use the formula of decision-making process.

$$A_{\tau j}^{\prime }=\left\{ \begin{array}{c} A_{\tau j\;}for\;benefit\;attribute\;\mu_{j}\\ A_{\tau j}^{c}\;for\;cost\;attribute\;\mu_{j} \end{array}\right.$$

$A_{\tau j}^{c}=\left(\lambda_{A\tau }\left(\mu_{j}\right),\tau_{A\tau }\left(\mu_{j}\right),d_{A\tau }^{\prime }\left(\mu_{j}\right)\right),\tau =\left\{1,2,\ldots n\right\},j=\left\{1,2\ldots m\right\}$. The above formula based on the alternative $A_{\tau }=\left\{A_{\tau j}^{\prime }\right\}$.

**Step 2:** The similarity measure $L=\left(A_{\tau },A\right)\left(\tau =1,2,3,\ldots ,n\right)$ where A={(0.99,0.79,0.22),(0.99,0.79,0.22),…,(0.99,0.79,0.22)} is an alternative with the attributes m.
**Step 3:** The maximum one is chosen in $L=\left(A_{\tau_{0}},A\right)$ from L=(Aτ,A)τ=(τ=1,2,3,…n)τ.eL=(Aτ0,A)={L(Aτ,A)}1≤τ≤nmaμ . Then the maximum similarity measures alternative $A_{\tau 0}$ according to the principle of maximum.
**Step 4:** The degree of ${Doc}^{\tau o}\sum_{\tau =1,\tau \neq \tau o}^{n}│L\left(A_{\tau_{0},}A\right)-L\left(A_{\tau },A\right)│{Doc}^{\tau_{0}}$ is the great result of similarity measure.

In the following example for the similarity measure ${\;L}_{m}\text{,}$ $P=3,m_{1}=m_{2}=m_{3}=3$.

**Example 2:** There are three alternatives $A_{1},A_{2},A_{3}$ with three different attributes $\mu_{1},\mu_{2},\mu_{3}$ described the SVNSs as shown in Table 3.

**Table 3:** Three alternatives with three attributes.

	$x_{1}$	$x_{2}$	$x_{3}$
$\varphi_{1}$	(0.99,0.78,0.79)	(0.67,0.99,0.55)	(0.93,0.68,0.98)
$\varphi_{2}$	(0.66,0.98,0.99)	(0.66,0.34,0.99)	(0.25,0.99,0.78)
$\varphi_{3}$	(0.77,0.89,0.68)	(0.99,0.89,0.86)	(0.93,0.98,0.68)

In the following, Table 4 shows the values of the SMs of ${\;A}_{1},A_{2}\text{,}$ and ${\;A}_{3}$ with A.

**Table 4:** Values of the Similarity Measures and Decision results of the Example 2.

	$L\left(\varphi_{1},\varphi \right)$	$L\left(\varphi_{2},\varphi \right)$	$L\left(\varphi_{3},\varphi \right)$
$L_{1}$	0.9041	0.758	0.9306
$L_{2}$	0.9828	0.8731	0.9786
$L_{3}$	0.9807	0.7758	0.9658
$L_{4}$	0.7378	0.5178	0.7522
$L_{5}$	0.5596	0.0563	0.6496
$L_{6}$	1.32	1.09	1.12
$L_{7}$	0.4467	0.24	0.48
$L_{8}$	0.6207	0.368	0.6787
$L_{9}$	0.9135	0.7253	0.9251
$L_{10}$	0.0281	−0.6012	−0.0536
$L_{11}$	0.5379	0.0811	0.534
$L_{12}$	0.9834	0.3961	0.9677
$L_{13}$	0.7427	0.9796	0.6957
$L_{m}$	0.3922	0.0063	0.357

Table 4 shows the values of the SMs for the alternatives based on the attributes. Now, we find the ranking of the alternatives based on the values obtained from the SMs. In the following, Table 6 shows the decision ranking of the alternatives.

**Table 5:** Ranking of the alternatives with the help of the values of the SMs obtained in Table 4.

	Ranking	The best alternative	${Doc}^{\left(3\right)}$
$L_{1}$	$\varphi_{3}>\varphi_{1}>\varphi_{2}$	$\varphi_{3}$	0.2922
$L_{2}$	$\varphi_{1}>\varphi_{3}>\varphi_{2}$	$\varphi_{1}$	0.2194
$L_{3}$	$\varphi_{1}>\varphi_{3}>\varphi_{2}$	$\varphi_{1}$	0.4098
$L_{4}$	$\varphi_{3}>\varphi_{1}>\varphi_{2}$	$\varphi_{3}$	0.4400
$L_{5}$	$\varphi_{3}>\varphi_{1}>\varphi_{2}$	$\varphi_{3}$	XXX
$L_{6}$	Does not satisfy	XXX	0.4600
$L_{7}$	$\varphi_{3}>\varphi_{1}>\varphi_{2}$	$\varphi_{3}$	0.4134
$L_{8}$	$\varphi_{3}>\varphi_{1}>\varphi_{2}$	$\varphi_{3}$	0.5054
$L_{9}$	$\varphi_{3}>\varphi_{1}>\varphi_{2}$	$\varphi_{3}$	0.3764
$L_{10}$	$\varphi_{1}>\varphi_{3}>\varphi_{2}$	$\varphi_{1}$	XXX
$L_{11}$	$\varphi_{1}>\varphi_{3}>\varphi_{2}$	$\varphi_{1}$	0.9136
$L_{12}$	$\varphi_{1}>\varphi_{3}>\varphi_{2}$	$\varphi_{1}$	XXX
$L_{13}$	$\varphi_{2}>\varphi_{1}>\varphi_{3}$	$\varphi_{2}$	XXX
$L_{m}$	$\varphi_{1}>\varphi_{3}>\varphi_{2}$	$\varphi_{1}$	0.2922

**Table 6:** Evaluation results of six faculty alternative in this example.

	$x_{1}$	$x_{2}$	$x_{3}$
$\varphi_{1}$	(0.85,0.77,0.99)	(0.93,0.83,0.89)	(0.77,0.99,0.55)
$\varphi_{2}$	(0.99,0.83,0.99)	(0.95,0.77,0.66)	(0.89,0.66,0.93)
$\varphi_{3}$	(0.56,0.78,0.99)	(0.77,0.66,0.98)	(0.78,0.93,0.86)
$\varphi_{4}$	(0.67,0.92,0.73)	(0.56,0.99,0.66)	(0.86,0.87,0.95)
$\varphi_{5}$	(0.67,0.98,0.94)	(0.98,0.77,0.65)	(0.98,0.78,0.67)
$\varphi_{6}$	(0.77,0.99,0.89)	(0.79,0.97,0.57)	(0.99,0.77,0.87)

**Fig. 1:**
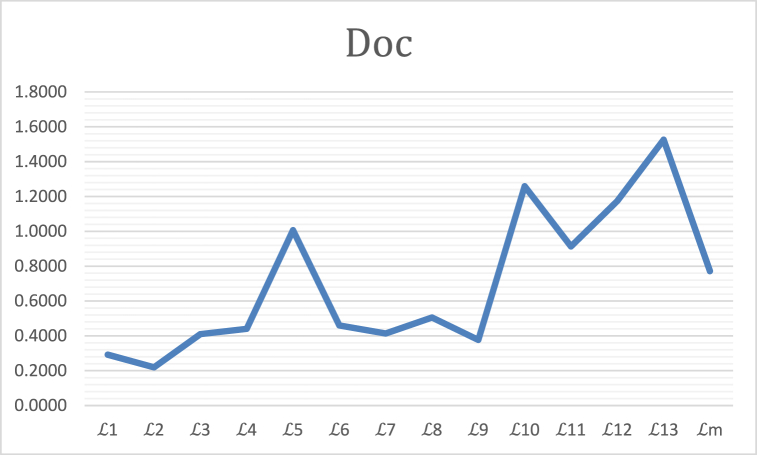
In this example the degree of ${Doc}^{\left(3\right)}$ confidence.

It is cleared from Table 5: the axiom $\varphi_{1}$ and $\varphi_{3}$ is one of the most basic axioms of picture SMs. By analyzing Table 5, we can easily find that the similarity measures but $L_{3}$ do not satisfy this axiom. The ranking of the alternatives is geometrically represented by Fig. 1 as follows.

In this case $\varphi_{1}$ and $\varphi_{3}$ is the best SMs results. And $L_{5},L_{10},L_{12}\;and\;L_{13}$ does not satisfy in the SMs. Because when we apply the formula ${Doc}^{\left(3\right)}$ then the answer $L_{5},L_{10},L_{12}\;and\;L_{13}$ not in [0,1]. The definition of ${Doc}^{\left(3\right)}$ given in step 4.

Example 3:

In this section we find the best away of energy production is a multifaceted process that encompasses various technologies and resources to generate electricity or other forms of energy. Traditional methods such as fossil fuel combustion, hydroelectric dams, and nuclear power plants have historically dominated the sector. However, the global shift towards sustainable practices has led to a significant increase in renewable energy production. Solar photovoltaic arrays harness sunlight, wind turbines convert wind energy into electricity, and biomass facilities utilize organic materials for power generation. Additionally, advancements in energy storage technologies, such as lithium-ion batteries, play a pivotal role in balancing the intermittent nature of renewables. The integration of smart grids and energy-efficient technologies further enhances the overall efficiency and reliability of energy production, fostering a transition towards a more sustainable and resilient energy landscape. We these are six $\left(\varphi_{1},\varphi_{2},\varphi_{3},\varphi_{4},\varphi_{5},\varphi_{6}\right)$ alternatives for energy production.

## 6 Alternative approaches to energy production

### 6.1 Hydrogen production via electrolysis $\varphi_{1}$

Explore the production of hydrogen through electrolysis, using renewable energy sources like wind or solar to split water into hydrogen and oxygen. Hydrogen can be used as a clean fuel for various applications.

### 6.2 Advanced nuclear reactors $\varphi_{2}$

Investigate advanced nuclear reactor designs, such as small modular reactors (SMRs) or thorium-based reactors, which offer potential improvements in safety, efficiency, and reduced nuclear waste compared to traditional nuclear power plants.

### 6.3 Enhanced geothermal systems (EGS) ${\;\varphi }_{3}$

Develop Enhanced Geothermal Systems that go beyond traditional geothermal power by fracturing hot rock formations deep underground, increasing the availability of geothermal energy resources in regions without natural geothermal reservoirs.

### 6.4 Solar thermal power plants $\varphi_{4}$

Deploy solar thermal power plants that use concentrated solar power to generate electricity. Mirrors or lenses focus sunlight onto a small area, producing high-temperature heat that can be used to generate steam and drive turbines.

### 6.5 Piezoelectric and kinetic energy harvesting ${\;\varphi }_{5}$

Implement technologies that harvest energy from ambient sources, such as piezoelectric materials that convert mechanical vibrations into electricity or kinetic energy harvesting systems that capture energy from movement.

### 6.6 Bioenergy with carbon capture and storage (BECCS) $\varphi_{6}$

Combine bioenergy with carbon capture and storage to create a negative carbon emissions process. Biomass is used to generate energy, and the resulting carbon dioxide emissions are captured and stored to prevent them from entering the atmosphere.

These alternative approaches contribute to the diversification of the energy mix and address specific challenges related to sustainability, efficiency, and environmental impact.

Now we choose in these alternatives $\left(\varphi_{1},\varphi_{2},\varphi_{3},\varphi_{4},\varphi_{5},\varphi_{6}\right)$ on these three $\left(\mu_{1},\mu_{2},\mu_{3}\right)$ attitudes.

### 6.7 Attitudes towards energy production

#### 6.7.1 Community-centric perspective $x_{1}$

Embrace a community-centric attitude, involving local communities in decision-making processes related to energy production to ensure that projects align with their needs, values, and aspirations.

#### 6.7.2 Resilience and reliability focus $x_{2}$

Prioritize resilience and reliability in energy production, emphasizing the need for robust infrastructure and diversified energy sources to ensure uninterrupted power supply, especially during unforeseen events or emergencies.

#### 6.7.3 Global collaboration stance $x_{3}$

Adopt a global collaboration stance, recognizing the interconnectedness of energy systems worldwide and promoting international cooperation to address common challenges, share knowledge, and collectively work towards a sustainable energy future.

**Fig. 2:**
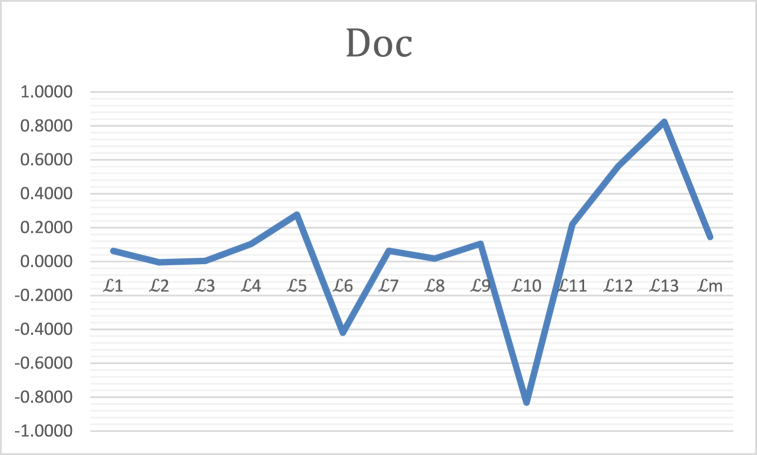
In this example the degree of ${Doc}^{\left(2\right)}$ confidence.

It is cleared from Table 8, in this case SMs on the e principle of maximum given the different SMs and the best SMs have different result seen in Table 7 and Fig. 2. But the $L_{2}$, $L_{3}{,L}_{4}{,L}_{5},L_{7},L_{8},L_{12}$ given the same result $\varphi_{5}$. And we apply the definition ${Doc}^{\left(2\right)}$ given in step 4. Some SMs $L_{6}$ and $L_{13}$ cannot satisfy the SMs result. And Some SMs $L_{2},L_{6}$ and $L_{10}$ given the negative answer when we apply the formula ${Doc}^{\left(2\right)}$.

**Table 7:** Similarity measures and decision results in this example.

L	The result of similarity measures					
	$L\left(\varphi_{1},\varphi \right)$	$L\left(\varphi_{2},\varphi \right)$	$L\left(\varphi_{3},\varphi \right)$	$L\left(\varphi_{4},\varphi \right)$	$L\left(\varphi_{5},\varphi \right)$	$L\left(\varphi_{6},\varphi \right)$
$L_{1}$	0.9007	0.8995	0.8323	0.8821	0.9156	0.9115
$L_{2}$	0.956	0.946	0.9078	0.9817	0.9874	0.9623
$L_{3}$	0.9293	0.9113	0.8316	0.9732	0.9883	0.939
$L_{4}$	0.7278	0.75	0.6322	0.67	0.76	0.7233
$L_{5}$	0.6403	0.6037	0.3987	0.4916	0.7695	0.6613
$L_{6}$	XXX	XXX	XXX	XXX	0.95	XXX
$L_{7}$	0.41	0.36	0.2767	0.44	0.4667	0.4433
$L_{8}$	0.5725	0.5213	0.4191	0.626	0.6555	0.6235
$L_{9}$	0.9092	0.9195	0.8368	0.8682	0.9136	0.9028
$L_{10}$	−0.3604	−0.2591	−0.554	0.0013	−0.1088	−0.0496
$L_{11}$	0.4809	0.5693	0.2847	0.3718	0.5041	0.4541
$L_{12}$	0.90048	0.6921	0.4694	0.8764	0.9632	0.9383
$L_{13}$	XXX	XXX	XXX	XXX	XXX	XXX
$L_{m}$	0.3041	0.435	0.1236	0.1452	0.383	0.2885

**Table 8:** The best alternative result.

	Ranking	The best alternative	${Doc}^{\left(2\right)}$
$L_{1}$	$\varphi_{6}>\varphi_{1}>\varphi_{5}>\varphi_{2}>\varphi_{4}>\varphi_{3}$	$\varphi_{6}$	0.0625
$L_{2}$	$\varphi_{5}>\varphi_{4}>\varphi_{6}>\varphi_{1}>\varphi_{2}>\varphi_{3}$	$\varphi_{5}$	−0.0052
$L_{3}$	$\varphi_{5}>\varphi_{4}>\varphi_{6}>\varphi_{1}>\varphi_{2}>\varphi_{3}$	$\varphi_{5}$	0.0031
$L_{4}$	$\varphi_{5}>\varphi_{2}>\varphi_{1}>\varphi_{6}>\varphi_{4}>\varphi_{3}$	$\varphi_{5}$	0.1035
$L_{5}$	$\varphi_{5}>\varphi_{6}>\varphi_{1}>\varphi_{2}>\varphi_{4}>\varphi_{3}$	$\varphi_{5}$	0.2767
$L_{6}$	Doesnotsatisfy	$\varphi_{6}$	−0.4200
$L_{7}$	$\varphi_{5}>\varphi_{6}>\varphi_{4}>\varphi_{2}>\varphi_{2}>\varphi_{3}$	$\varphi_{5}$	0.0633
$L_{8}$	$\varphi_{5}>\varphi_{6}>\varphi_{4}>\varphi_{1}>\varphi_{2}>\varphi_{3}$	$\varphi_{5}$	0.0171
$L_{9}$	$\varphi_{2}>\varphi_{5}>\varphi_{1}>\varphi_{6}>\varphi_{3}>\varphi_{4}$	$\varphi_{2}$	0.1051
$L_{10}$	$\varphi_{3}>\varphi_{5}>\varphi_{6}>\varphi_{2}>\varphi_{1}>\varphi_{3}$	$\varphi_{3}$	−0.8318
$L_{11}$	$\varphi_{2}>\varphi_{5}>\varphi_{1}>\varphi_{6}>\varphi_{4}>\varphi_{3}$	$\varphi_{2}$	0.2205
$L_{12}$	$\varphi_{5}>\varphi_{6}>\varphi_{1}>\varphi_{4}>\varphi_{2}>\varphi_{3}$	$\varphi_{5}$	0.5630
$L_{13}$	Doesnotsatisfy	$\varphi_{3}$	0.8241
$L_{m}$	$\varphi_{1}>\varphi_{5}>\varphi_{1}>\varphi_{6}>\varphi_{3}>\varphi_{4}$	$\varphi_{1}$	0.1452

The ranking of the alternatives is geometrically represented by Fig. 2 as follows.

#### 6.7.4 Analysis and discussion

Through the analysis of the mathematical experiment and MADM problem, the viability and adaptability of the proposed SM are shown. There are a few advances that can't be overlooked when contrasted with the some current similarity measures $L_{1}$, $L_{2}$, $L_{3}$, $L_{4},L_{5},L_{6}L_{7},L_{9},L_{10}$ $L_{11}$ $L_{12},L_{13},and\;L_{m}$.

(1) With the assistance of the current similarity measures, decision makers focus on the positioning of dynamic other options, however they overlook the impact of decision maker's style and choice climate on dynamic outcomes. The similarity measure proposed in this paper expands its adaptability by presenting boundaries $m_{1},m_{2},m_{3}$. For this situation, decision makers can took for the proper boundaries $m_{1},m_{2},m_{3}$ to get the sensible similarity measure, which is in accordance with the ongoing leader style and choice climate. For more to this data see [75, 76].
(2) The proposed SMs fulfills the maxim (S2) of the similarity measure, which makes the proposed SMs stay away from the circumstance of counter instinct A=B infers L(A,B)=1. Furthermore, the proposed similarity measure expands the distinguishing ability by containing a ton of Neutrosophic in line and defeats the imperfections of some current similarity measures ${\;L}_{1}$, $L_{2}$, $L_{3}$, $L_{4},L_{5},L_{6}L_{7},L_{9},L_{10}$ $L_{11}$ $L_{12},L_{13},L_{m}$.
(3) The proposed SMs can give sensible dependable choice results to leaders. The proposed similarity measure has the most significant level of believability, yet in addition can take care of the dynamic issues that the current SMs can't settle and get sensible choice outcomes. Hence, the proposed SMs is sensible and adaptable. For more to this data see [77, 78].

## 7 Conclusions

While some similarity measures between SVNSs have been proposed, most of them have produced absurd results. In this paper, we first investigated and explained the causes of irrational results, and then we developed a new parametric similarity measure between SVNSs that can overcome some of the drawbacks of existing SVNSs similarity measures. We conclude that the suggested similarity measure is reasonable and consistent with human perception based on a detailed analysis and correlation between the suggested similarity measures and the existing similarity measures in Table 1, Table 2, Table 3 Based on the decisions made in Examples 1 through 3, it can be concluded that the suggested similarity measure produces the desired results. Ultimately, compared to some existing similarity measures between SVNSs for multi-characteristic independent direction, the proposed similarity measure makes the most sense for comparability. Not only does it have the best possible arrangement for decision-making, but it also has the highest possible standard. Furthermore, decision makers can be choose in different decision styles by adjusting the boundary $m_{1},m_{2},m_{3}$ of the proposed similarity measure in a wide assortment of decision making situations. Later on, we will focus on the relationship between the entropy, distance, and similarity measure between SVNSs and TSNSs.

## 8 Future direction

Later on, we will focus on the relationship between the entropy, distance, and similarity measures between SVNSs. As these are advanced topics one can also elaborate to study these, and the article given in the [[72], [73], [74]] also considered as a debate of discussion in the future work.

## 9 Limitation of the proposed model

It is worth noting that the results of this paper can be extended to the hesitant fuzzy environment. The SVNSs is limited only when the scenario of objects can't cover the domain outside 0 to 1. The proposed parametric similarity measure for neutrosophic sets has some limitations, including sensitivity to parameter selection and dependence on expert input for set construction. Additionally, its computational complexity and scalability in high-dimensional datasets require further investigation. These limitations underscore the need for future research to enhance the measure's robustness and applicability in energy production contexts.

## Data and code availability statement

Data will be made available on request.

## CRediT authorship contribution statement

**Peide Liu:** Writing – review & editing, Writing – original draft, Funding acquisition, Data curation, Conceptualization. **Muhammad Azeem:** Methodology, Investigation, Formal analysis. **Mehwish Sarfraz:** Writing – review & editing, Writing – original draft, Resources, Project administration. **Senesie Swaray:** Writing – review & editing, Writing – original draft, Visualization, Validation, Supervision, Software. **Bandar Almohsen:** Writing – review & editing, Writing – original draft, Supervision, Software.

## Declaration of competing interest

Authors declare no conflict of interest.

## References

[1] Wang H., Smarandache F., Zhang Y., Sunderraman R. (2010). Single valued neutrosophic sets. Multispace Multistructure.

[2] Zadeh L.A. (1965). Fuzzy sets. Inf. Control.

[3] Atanassov K.T. (1986). Intuitionistic fuzzy sets. Fuzzy Set Syst..

[4] Atanassov K.T. (1994). Operators over interval valued intuitionistic fuzzy sets. Fuzzy Set Syst..

[5] Smarandache F. Neutrosophy (1998).

[6] Ye J.A. (2014). Multicriteria decision-making method using aggregation operators for simplified neutrosophic sets. J. Intell. Fuzzy Syst..

[7] Smarandache F. (2019). Neutrosophic set is a generalization of intuitionistic fuzzy set, inconsistent intuitionistic fuzzy set (picture fuzzy set, ternary fuzzy set), pythagorean fuzzy set, spherical fuzzy set, and q-rung Orthopair fuzzy set, while neutrosophication is a generalization of regret theory, grey system theory, and three-ways decision (revisited). JNT (J. Neural Transm.).

[8] Garg H. (2018). Nancy new logarithmic operational laws and their applications to multiattribute decision making for single-valued neutrosophic numbers. Cognit. Syst. Res..

[9] Chi P., Liu P. (2013). An extended TOPSIS method for the multiple attribute decision making problems based on interval neutrosophic set. Neutrosophic Sets and Systems.

[10] Nagarajan D., Gobinath V.M., Broumi S. (2023). Multicriteria decision making on 3D printers for economic manufacturing using neutrosophic environment. Neutrosophic Sets and Systems.

[11] Ye J. (2011). Cosine similarity measures for intuitionistic fuzzy sets and their applications. Math. Comput. Model..

[12] Ye J. (2014).

[13] Ye J. (2014). Clustering methods using distance-based similarity measures of single-valued neutrosophic sets. J. Intell. Syst..

[14] Ye J. (2015). Improved cosine similarity measures of simplified neutrosophic sets for medical diagnoses. Artif. Intell. Med..

[15] Ali Z., Mahmood T. (2020). Complex neutrosophic generalised Dice similarity measures and their application to decision making. CAAI Transactions on Intelligence Technology.

[16] Liu P., Chu Y., Li Y., Chen Y. (2014). Some generalized neutrosophic number hamacher aggregation operators and their application to group decision making. Int. J. Fuzzy Syst..

[17] Saqlain M., Riaz M., Saleem M.A., Yang M.-S. (2021). Distance and similarity measures for neutrosophic hypersoft set (NHSS) with construction of NHSS-TOPSIS and applications. IEEE Access.

[18] Ye J., Du S. Some Distances (2019). Similarity and entropy measures for interval-valued neutrosophic sets and their relationship. Int. J. Mach. Learn. & Cyber.

[19] Ye J. (2021). Similarity measures based on the generalized distance of neutrosophic Z-number sets and their multi-attribute decision making method. Soft Comput..

[20] Chai J.S., Selvachandran G., Smarandache F., Gerogiannis V.C., Son L.H., Bui Q.-T., Vo B. (2021). New similarity measures for single-valued neutrosophic sets with applications in pattern recognition and medical diagnosis problems. Complex Intell. Syst..

[21] Peng X., Smarandache F. (2020). New multiparametric similarity measure for neutrosophic set with big data industry evaluation. Artif. Intell. Rev..

[22] Şahin R., Karabacak M. (2020). Optimization Theory Based on Neutrosophic and Plithogenic Sets.

[23] Sarfraz M., Pamucar D. (2024). A parametric similarity measure for spherical fuzzy sets and its applications in medical equipment selection. J. Eng. Manag. Syst. Eng.

[24] Bui Q.-T., Ngo M.-P., Snasel V., Pedrycz W., Vo B. (2023). Information measures based on similarity under neutrosophic fuzzy environment and multi-criteria decision problems. Eng. Appl. Artif. Intell..

[25] Thao N.X., Smarandache F. (2020). Apply new entropy based similarity measures of single valued neutrosophic sets to select supplier material. J. Intell. Fuzzy Syst..

[26] Ali M., Hussain Z., Yang M.-S. (2022). Hausdorff distance and similarity measures for single-valued neutrosophic sets with application in multi-criteria decision making. Electronics.

[27] Özlü Ş., Karaaslan F. (2022). Hybrid similarity measures of single-valued neutrosophic type-2 fuzzy sets and their application to MCDM based on TOPSIS. Soft Comput..

[28] Mondal K., Pramanik S., Giri B.C. (2020). Optimization Theory Based on Neutrosophic and Plithogenic Sets.

[29] Borah G., Dutta P. (2021). Multi-attribute cognitive decision making via convex combination of weighted vector similarity measures for single-valued neutrosophic sets. Cogn Comput.

[30] Mandour S. (2023). An exhaustive review of neutrosophic logic in addressing image processing issues. Neutrosophic Systems with Applications.

[31] Chaira T. (2022). Neutrosophic set based clustering approach for segmenting abnormal regions in mammogram images. Soft Comput..

[32] Bakro M., Al-Kamha R., Kanafani Q.A. (2020). Neutrosophic approach to digital images. Neutrosophic Sets and Systems.

[33] Kaur G., Garg H. (2022). A new method for image processing using generalized linguistic neutrosophic cubic aggregation operator. Complex Intell. Syst..

[34] Poonia M., Bajaj R.K., Singh K. (2022).

[35] Luo J., Zhuo W., Liu S., Xu B. (2024). The optimization of carbon emission prediction in low carbon energy economy under big data. IEEE Access.

[36] Zhu C., Zhang Y., Wang M., Deng J., Cai Y., Wei W., Guo M. (2024). Simulation and comprehensive study of a new trigeneration process combined with a gas turbine cycle, involving transcritical and supercritical CO2 power cycles and Goswami cycle. J. Therm. Anal. Calorim..

[37] Mondal K., Pramanik S. (2015). Neutrosophic tangent similarity measure and its application to multiple attribute decision making. Neutrosophic sets and systems.

[38] El-Shorbagy M.A., Nabwey H.A., Inc M., Khater M. (2023). A review on metaheuristic algorithms with neutrosophic sets for image enhancement. International Journal of Neutrosophic Science (IJNS).

[39] Song S., Jia Z., Yang J., Kasabov N.K. (2020). A fast image segmentation algorithm based on saliency map and neutrosophic set theory. IEEE Photon. J..

[40] Thong P.H., Smarandache F., Tuan T.M., Ngan T.T., Thai V.D., Giang N.L., Son L.H. (2023). Picture-neutrosophic trusted safe semi-supervised fuzzy clustering for noisy data. Comput. Syst. Sci. Eng..

[41] Mandour S., Ahmed K. (2021). Neutrosophic sets integrated with metaheuristic algorithms: a survey. Neutrosophic Sets and Systems.

[42] Dey A., Kumar R., Broumi S., Bhowmik P. (2022). Different types of operations on neutrosophic graphs. International Journal of Neutrosophic Science (IJNS).

[43] Ma K., Yang J., Liu P. (2020). Relaying-assisted communications for demand response in smart grid: cost modeling, game strategies, and algorithms. IEEE J. Sel. Area. Commun..

[44] Shirkhani M., Tavoosi J., Danyali S., Sarvenoee A.K., Abdali A., Mohammadzadeh A., Zhang C. (2023). A review on microgrid decentralized energy/voltage control structures and methods. Energy Rep..

[45] Zhu C., Zhang Y., Wang M., Deng J., Cai Y., Wei W., Guo M. (2024). Optimization, validation and analyses of a hybrid PV-battery-diesel power system using enhanced electromagnetic field optimization algorithm and ε-constraint. Energy Rep..

[46] Jafar M.N., Saeed M., Saqlain M., Yang M.-S. (2021). Trigonometric similarity measures for neutrosophic hypersoft sets with application to renewable energy source selection. IEEE Access.

[47] Hussain A., Pamucar D. (2022). Multi-attribute group decision-making based on pythagorean fuzzy rough set and novel schweizer-sklar T-norm and T-conorm. Journal of Innovative Research in Mathematical and Computational Sciences.

[48] Sarfraz M. (2024). Application of interval-valued T-spherical fuzzy dombi hamy mean operators in the antiviral mask selection against COVID-19. Journal of Decision Analytics and Intelligent Computing.

[49] Sarfraz M. (2024). Maclaurin symmetric mean aggregation operators based on novel Frank T-norm and T-conorm for picture fuzzy multiple-attribute group decision-making. Decision Making Advances.

[50] Sarfraz M. (2024). A few maclaurin symmetric mean aggregation operators for spherical fuzzy numbers based on schweizer-sklar operations and their use in. Artif. Intell..

[51] Sarfraz M. (2024). Multi-attribute decision-making for T-spherical fuzzy information utilizing schweizer-sklar prioritized aggregation operators for recycled water. Decision Making Advances.

[52] Chou S., Duong T.T.T., Thao N.X. (2021). Renewable energy selection based on a new entropy and dissimilarity measure on an interval-valued neutrosophic set. J. Intell. Fuzzy Syst..

[53] Eroğlu H., Şahin R. (2020). A neutrosophic VIKOR method-based decision-making with an improved distance measure and score function: case study of selection for renewable energy alternatives. Cogn Comput.

[54] Sarfraz M., Ullah K., Akram M., Pamucar D., Božanić D. (2022). Prioritized aggregation operators for intuitionistic fuzzy information based on aczel–alsina T-norm and T-conorm and their applications in group decision-making. Symmetry.

[55] Hussain A., Zhu X., Ullah K., Sarfaraz M., Yin S., Pamucar D. (2023). Multi-attribute group decision-making based on pythagorean fuzzy rough aczel-alsina aggregation operators and its applications to medical diagnosis. Heliyon.

[56] Ullah K., Sarfraz M., Akram M., Ali Z., Jana C., Pal M., Muhiuddin G., Liu P. (2023). Fuzzy Optimization, Decision-Making and Operations Research.

[57] Sarfraz M. (2024). Aczel-alsina aggregation operators on spherical fuzzy rough set and their application section of solar panel. J. Oper. Strateg Anal..

[58] Pramanik S., Biswas P., Giri B.C. (2017). Hybrid vector similarity measures and their applications to multi-attribute decision making under neutrosophic environment. Neural Comput. Appl..

[59] Boran F.E., Akay D. (2014). A biparametric similarity measure on intuitionistic fuzzy sets with applications to pattern recognition. Inf. Sci..

[60] Du W.S., Hu B.Q. (2015). Aggregation distance measure and its induced similarity measure between intuitionistic fuzzy sets. Pattern Recogn. Lett..

[61] Donyatalab Y., Farrokhizadeh E., Shishavan S.A.S. (2020). Proceedings of the International Conference on Intelligent and Fuzzy Systems.

[62] Mohd W.R.W., Abdullah L. (2018).

[63] Wei G. (2017). Some cosine similarity measures for picture fuzzy sets and their applications to strategic decision making. Informatica.

[64] Wei G., Gao H. (2018). The generalized Dice similarity measures for picture fuzzy sets and their applications. Informatica.

[65] Van Dinh N., Xuân Thảo N. (2018). Some measures of picture fuzzy sets and their application in multi-attribute decision making. Int. J. Math. Soft Comput..

[66] Risk analysis of flood disaster based on similarity measures in picture fuzzy environment | SpringerLink. https://link.springer.com/article/10.1007/s13370-018-0597-x.

[67] Luo M., Zhang Y. (2020). A new similarity measure between picture fuzzy sets and its application. Eng. Appl. Artif. Intell..

[68] Zhao R.R., Luo M.X., Li S.G., Ma L.N. (2023). A parametric similarity measure between picture fuzzy sets and its applications in multi-attribute decision-making. Iranian Journal of Fuzzy Systems.

[69] Ye J. (2014). Single valued neutrosophic cross-entropy for multicriteria decision making problems. Appl. Math. Model..

[70] Ye J., Fu J. (2016). Multi-Period medical diagnosis method using a single valued neutrosophic similarity measure based on tangent function. Comput. Methods Progr. Biomed..

[71] Ye J., Cui W. (2018). Exponential entropy for simplified neutrosophic sets and its application in decision making. Entropy.

[72] Zhu C. (2023). An adaptive agent decision model based on deep reinforcement learning and autonomous learning. Journal of Logistics, Informatics and Service Science.

[73] Meng Q., Tong X., Hussain S., Luo F., Zhou F., Liu L., Li B. (2024). Revolutionizing photovoltaic consumption and electric vehicle charging: a novel approach for residential distribution systems. IET Gener., Transm. Distrib..

[74] Meng Q., Jin X., Luo F., Wang Z., Hussain S. (2024). Distributionally robust scheduling for benefit allocation in regional integrated energy system with multiple stakeholders. Journal of Modern Power Systems and Clean Energy.

